# Clinical and Translational Science Award hubs in learning health systems: evaluation framework of the engine-drivetrain model

**DOI:** 10.1186/s12967-026-08123-z

**Published:** 2026-04-22

**Authors:** Octavian C. Ioachimescu

**Affiliations:** Department of Medicine, Division of Pulmonary, Critical Care and Sleep Medicine, Medical College of Wisconsin, Froedtert ThedaCare, and Clement J. Zablocki Veteran Affairs Medical Center, Milwaukee, Wisconsin USA

## Abstract

**Background:**

Learning Health Systems (LHS) aim to accelerate generation, implementation, and dissemination of knowledge to improve population health. Community engagement is widely recognized as an essential component of LHS, yet methods for evaluating community empowerment and its impact on translational performance remain limited. Most assessments focus on participation metrics rather than structural influence on governance, research, and care delivery.

**Results:**

We proposed a conceptual and quantitative evaluation framework in which the Clinical and Translational Science Award (CTSA) hub serves as the translational science engine of the system, while community empowerment serves as the axle and the variable transmission (drivetrain) regulating the conversion of institutional resources into translational performance. System outputs are expressed through four coupled LHS cycles (clinical care, education, research, and governance), each characterized by translational velocity, innovation throughput, and economic performance. Community empowerment is quantified using the Community Transmission Index (CTI), a structured instrument that evaluates engagement maturity across eight domains including shared governance, participatory data governance, co-production, trust capital, and equity integration. The CTI generates a standardized 0–1 score that reflects the extent to which community partners shape institutional decision-making and system operations. Translational performance across the four LHS cycles can be evaluated using measures such as velocity (inverse latency to sustainment), innovation output, return on investment, and translational efficiency indices. We hypothesized that higher CTI scores will increase translational velocity, improve balance across learning cycles, and enhance innovation uptake by reducing sociotechnical friction and strengthening the legitimacy and sustainability of change processes.

**Conclusions:**

This framework offers a structured approach for evaluating how community empowerment influences translational performance in LHS and how it can be assessed using complementary metrics. By linking engagement measures to operational and economic outcomes, the model enables LHS and academic institutions to assess whether community partnerships function as advisory mechanisms or as integral components of the translational infrastructure.

**Supplementary Information:**

The online version contains supplementary material available at 10.1186/s12967-026-08123-z.

## Key messages

What is Known

What is the Gap

What is Novel Here

•Learning Health Systems (LHS) integrate clinical care, research, education, and governance into iterative cycles that accelerate translation of knowledge into improved health outcomes.

•Clinical and Translational Science Award (CTSA) hubs can provide the core infrastructure (methods, informatics and biostatistics, pilot funding, training programs, and implementation science capabilities) that powers the system, enabling LHS members to generate knowledge and implement innovations.

•Community engagement is widely recognized as critical for ensuring relevance, trust, equity, and adoption of innovations in both LHS and CTSA activities.

•Traditional engagement metrics largely measure participation or process indicators (e.g., advisory boards, meetings, stakeholder involvement).

•Current frameworks rarely measure community empowerment (CE) as a structural influence on decision-making, governance, or system operations.

•Engagement metrics are seldom linked to translational performance or economic impact at the system level.

•Health systems often lack tools to determine whether community engagement is transactional, advisory, or truly transformative.

•We introduced a translational engine-drivetrain model in which the CTSA hub functions as the engine, CE operates as the axle and variable transmission, and four learning cycles of clinical care, education, research, and governance serve as the LHS’ wheels (drivetrain) Ioachimescu (J Transl Med, 2026).

•CE is neither a recruitment tool, nor a dissemination activity; rather, it is a structural component of the translational ecosystem, whose maturity determines translational efficiency and equity performance.

•We propose the use of Community Transmission Index (CTI), a novel structured instrument that measures CE across eight domains including shared governance, participatory data governance, co-production, trust capital, and equity integration.

•By linking CTI scores to translational velocity and efficiency, innovation throughput, and return on investment, the framework enables evaluation of how CE affects the performance and sustainability of LHS.

**Supplementary Information:**

The online version contains supplementary material available at 10.1186/s12967-026-08123-z.

## Introduction

Learning Health Systems (LHS) have emerged as a dominant paradigm for improving healthcare quality, accelerating research translation, and fostering continuous system improvement [1, 2]. In an LHS, clinical practice, research, education, and organizational governance operate in interconnected feedback loops that enable rapid learning from real-world data and experience. The central premise of the LHS model is that knowledge generation and implementation should occur simultaneously and iteratively, allowing healthcare systems to learn from every patient interaction and continuously refine care delivery.

Clinical and Translational Science Award (CTSA) programs were established in the United States to accelerate the translation of scientific discoveries into health improvements [3]. The CTSA hubs provide the methodological expertise, informatics infrastructure, and collaborative networks required to support translational research across institutions and communities. Within the LHS paradigm, CTSA hubs often function as institutional engines of translational science, supplying the power that drives research, innovation, and implementation activities [1, 4].

Despite active development and availability of translational infrastructures, the pace at which scientific discoveries are translated into improved patient care and population health remains slower than desired. Unfortunately, adequate engine power alone does not guarantee motion, and translation often stalls at adoption, scale, sustainment, or policy levels, domains that depend on legitimacy, trust, power sharing, and accountability. We previously proposed [1] a modified conceptualization of LHS as a four-cycle (or four-wheel) model centered by its community and strengthened by external evidence and data as distinct inputs at multiple points in the cycle. In this model, CTSA hub structures and functions represent the strong operational engine [4, 5], while community represents the central axle and variable transmission that governs motion in the core LHS mechanism [1].

Another major contributor to this gap is the insufficient integration of community perspectives into clinical care, research, education, governance, and implementation processes. Communities - including patients, family members, caregivers, advocacy groups, and other local stakeholders - play a crucial role in shaping health priorities, influencing adoption of innovations, and sustaining health system improvements. When community voices are excluded from decision-making, interventions may fail to address real-world needs, resulting in limited adoption and impact. We therefore view the community not as an external constituency but as the central axle that couples LHS cycles and as a variable transmission that regulates translational torque and speed. To the classic triad of missions (clinical, education and research cycles), we added a fourth wheel: governance, where rules, permissions, incentives, data rights, resources and institutional commitments are devised and implemented, which is essential for equity and sustainability as core system properties. 

This article presents a comprehensive conceptual and quantitative evaluative framework, along with a mathematical model for the recently developed LHS-CTSA engine-drivetrain model [1].

## Methods

The proposed model conceptualizes a LHS using an engine-drivetrain analogy that links translational capacity to system performance [1]. Its main components include:

- The CTSA hub as the translational engine, providing the system’s available translational power or torque through methods expertise, informatics infrastructure, workforce development, pilot funding, and convening capacity;
- The community as the axle, ensuring alignment and stability across cycles through legitimacy, trust, representativeness, and accountability;
- The community engagement or community empowerment (CE), which determines how power is shared and how feedback loops close. CE maturity converts CTSA power into system motion while reducing socio-structural friction. In this framework it functions as a variable transmission, operationalized as the Community Transmission Index (CTI);
- Four LHS operational cycles (“wheels”), representing core domains of system function:
  - Clinical care: evidence → care → outcomes → feedback → adaptation;
  - Education: education and training → practice → outcomes evaluation → curriculum refinement);
  - Research: innovation or scientific discovery → development → implementation → dissemination → policy;
  - Governance: leadership alignment → policy development → policy implementation → change management and leadership → policy re-evaluation → adaptation.

Although the CTSA hub acts as the engine of discovery and implementation, engines alone do not determine system motion. Torque must be transmitted efficiently to the wheels that interact with real-world institutional or community conditions. In the engine-drivetrain model, community is not conceptualized merely as a set of external stakeholders; rather, it functions as the central shaft coupling the system’s operational cycles and modulating the speed and distribution of translational energy. Low CE maturity results in energy loss, where interventions stall, adaptation is resisted, and equity gaps persist. Conversely, high CE maturity improves transmission efficiency, accelerates feedback loops, and reduces socio-structural friction. This relationship is formalized through the **Community Transmission Index (CTI),** which links CE maturity to translational velocity using time-to-event hazard modeling and mechanistic friction-reduction functions.

A LHS can be evaluated (Table 1) by quantifying **translational velocity** across its four core cycles: clinical care (V₁), education (V₂), research (V₃), and governance (V₄), and linking these to innovation outputs (N₁–N₄), revenues (R₁–R₄), expenses (E₁–E₄), and resulting return on investment (ROI). Each cycle’s velocity is defined as inverse of latency to scale or sustainment (measured in months). Velocities may be normalized using institutional or external percentile benchmarks, enabling cross-domain and cross-hub comparisons.

**Table 1:** Performance assessment of the 4-wheel LHS - Velocity metrics, operational definitions, numerators, denominators, and data provenance

LHS Cycle (Wheel)	Performance Construct	Primary metric/function (recommended)	Operational definition (numerator, denominator)	Secondary metrics (examples)	Data provenance
Clinical Care (1)	Care Learning Speed (Translational Velocity V_1_)	Clinical feedback latency	Median days from community or patient signal logged → clinical workflow change implemented (and confirmed)	V_1_min; V_1_max; institutionally scaled V_1_ = (V_1_ – Q_1_5^th^)/(Q_1_95^th^ - Q_1_5^th^); median time to scale/sustainment (months); stage-specific latency (e.g., assemble a transdisciplinary QI team in a specific clinic area)	EHR & QI systems, patient advisory inputs, issue trackers, CTSA Evaluation group and QI committee tracking
	# Innovations Implemented per year (Clinical)	N_1_		Adoption penetration (% sites); # PDCA cycles/quarter; change in community-prioritized PROs (patient related outcomes); % projects with community co-leads	
	Clinical Revenue (R_1_)	Revenue Function (V_1_, N_1_)			QI systems, Health System Finance Department
	Expense (E_1_)	Cost Function (V_1_, PDCA_p_, PDCA_d_, PDCA_i_)			
	Incremental Expense (ΔE_1_)	Incremental Cost Function (ΔV_1_)			
	Return on Investment (ROI_1_)	R_1_/E_1_, N_1_/E_1_, ΔV_1_/ΔE_1_,			
Education (2)	Workforce Learning Speed (Translational Velocity V_2_)	Curriculum adaptation latency	Median days from community-identified competency gap → curriculum revision approved + implemented	V_2_min; V_2_max; institutionally scaled V_2_ = (V_2_ – Q_2_5^th^)/(Q_2_95^th^ - Q_2_5^th^); median time to scale/sustainment (months); stage-specific latency (e.g., curriculum redesign team role assignments)	Curriculum committee logs, Learning Management System (LMS) timestamps, Human Resources (HR) – placement and retention data
	# Innovations Implemented per year (Education)	N_2_		% programs with community faculty; trainee competency attainment rate; placement/retention in community settings	
	Education Revenue (R_2_)	Revenue Function (V_2_, N_2_)			QI systems, Academic System Finance Department
	Expense (E_2_)	Cost Function (V_2_, PDCA_p_, PDCA_d_, PDCA_i_)			
	Incremental Expense	Incremental Cost Function (ΔV_2_)			
	Return on Investment (ROI_2_)	R_2_/E_2_, N_2_/E_2_, ΔV_2_/ΔE_2_,			
Research (3)	Discovery-to-use Speed (Translational Velocity V_3_)	Time-to-scale (research-to-implementation)	Median months from pilot start → scale decision or sustained adoption (predefined milestone)	V_3_min; V_3_max; institutionally scaled V_3_ = (V_3_ – Q_3_5^th^)/(Q_3_95^th^ - Q_3_5^th^); median time to scale/sustainment (months), stage-specific latency (e.g., IRB → first patient → pilot → scale) IRB-to-first-enrollment; return-of-results latency;	CTSA tracking, Institutional Review Boards (IRB) logs, Office of Research -grants, dissemination logs
	# Innovations Implemented per year (Research)	N_3_		% studies with community co-PIs	
	Research Revenue (R_3_)	Revenue Function (V_3_, N_3_)			QI systems, Academic System Finance Department
	Expense (E_3_)	Cost Function (V_3_, PDCA_p_, PDCA_d_, PDCA_i_)			
	Incremental Expense	Incremental Cost Function (ΔV_3_)			
	Return on Investment (ROI_3_)	R_3_/E_3_, N_3_/E_3_, ΔV_3_/ΔE_3_			
Governance (4)	Policy or Incentive Learning Speed (Translational Velocity V_4_)	Policy adaptation latency	Median days from validated community signal/evidence → policy/rule/incentive change enacted	V_4_min; V_4_max; institutionally scaled V_4_ = (V_4_ – Q_1_5^th^)/(Q_4_95^th^ - Q_4_5^th^); median time to scale/sustainment (months), stage-specific latency (e.g., leadership recruitment → development → alignment → impact)	Board minutes, policy registry, financial records, charters
	# Innovations Implemented per year (Leadership)	N_4_ (e.g., business model innovations)		% governance decisions using community evidence or input; resource allocation shifts; # new incentives or compensation policies; trust index; disparity index	
	Leadership Revenue (R_4_)	Revenue Function (V_4_, N_4_)			QI systems, Health System and Academic System Finance Departments
	Expense (E_4_)	Cost Function (V_4_, PDCA_p_, PDCA_d_, PDCA_i_)			
	Incremental Expense	Incremental Cost Function (ΔV_4_)			
	Return on Investment (ROI_4_)	R_4_/E_4_, N_4_/E_4_, ΔV_4_/ΔE_4_			
Global	Global Translational Velocity (V)	Median time to scale/sustainment (months) V = aV_1_ + bV_2_ + cV_3_ + dV_4_ + e [Colinearities between V_1_ , V_2_ , V_3_, V_4_ illustrate existing synergies] or V = ANN (V_1_ , V_2_ , V_3_, V_4_)		Bottleneck speed (drivetrain-limiting wheel) for a project p: ${v_{p,{\rm{sys}}}}\left( t \right) = {\rm{mi}}{{\rm{n}}_j}{v_{p,j}}\left( t \right)$ Translational Efficiency Index- Bottleneck (TEI-B): ${{{{\rm{v}}_{{\rm{p}},{\rm{min}}}}\left( {\rm{t}} \right)} \over {\mathop \sum \nolimits_{{\rm{j}} = 1}^4 {{\rm{\alpha }}_{\rm{j}}}{{\rm{v}}_{{\rm{p}},{\rm{j}}}}\left( {\rm{t}} \right)}}$ Weighted mean speed (mission-aligned) for a project p: ${v_{p,{\rm{sys}}}}\left( t \right) = \mathop \sum \limits_j {\alpha _j}{v_{p,j}}\left( t \right)$, $\mathop \sum \nolimits {\alpha _j} = 1$ Translational Efficiency Index- Gain (TEI-G) and TEI-G_hub_ = median_p,t_(TEI-G_p_(t)) Global TEI = TEI-G x TEI-B Cross-CTSA consortium (nationally) scaled V = (V - N5^th^)/(N95^th^ - N5^th^).	QI systems, Board minutes, policy registry, finance records, charters
	Global # Innovations implemented per year	*N* = N_1_ + N_2_ + N_3_ + N_4_			
	Global Revenue (R)	*R* = R_1_ + R_2_ + R_3_ + R_4_			Health System and Academic System Finance Departments
	Global Expense (E)	Cost Function (V_1_, V_2_, V_3,_ V_4_)			
	Global Incremental Expense (ΔE)	Incremental Cost Function (ΔV_1_, ΔV_2_, ΔV_3_, ΔV_4_)			
	Global Return on Investment (ROI)	R/E, ΔV/ΔE, N/ΔE			

Wheel velocities ${{\rm{v}}_p}\left( t \right) = {\left[ {{v_{p,1}}{\rm{,}}{v_{p,2}}{\rm{,}}{v_{p,3}}{\rm{,}}{v_{p,4}}} \right]^{}}$are computed per project $p$ per time window (monthly/quarterly). Use inverse time (e.g., $1/{\rm{\Delta }}t$) and/or throughput (events per unit time). Include at least one metric per wheel that is closed-loop (signal → action → confirmation).${v_{p,{\rm{sys}}}}\left( t \right):$global or system-level translational velocity at time t; ${\rm{mi}}{{\rm{n}}_j}{v_{p,j}}\left( t \right)$: the lowest speed of the LHS_j_ during project p at the time t; $\mathop \sum \limits_j {\alpha _j}{v_{p,j}}\left( t \right)$, $\mathop \sum \limits_j {\alpha _j}=1$: weighted average of various stage speeds in LHS_j_ during project p; $j \in \left( {0,1,2,3,4} \right)$

*Abbreviations:* a, b, c, d, e: Linear Regression coefficients; ANN: Artificial Neural Networks; E: Expense (cost); CTSA: Clinical and Translational Science Award hub; EHR: Electronic Health Records; HR: Human Resources; IRB: Institutional Review Board; LHS: Learning Health System; LMS: Learning Management System; PDCA: Plan-Do-Check-Act cycle; PDCA_d_: PDCA for the data analysis phase; PDCA_i_: PDCA for the implementation phase; PDCA_p_: PDCA for the practice phase; N5^th^ & N95^th^: 5^th^ & 95^th^ national percentiles of global LHS translational velocities; QI: Quality Improvement; Q_i_5^th^ & Q_i_95^th^: 5^th^ & 95^th^ institutional percentiles of project speeds in LHS of i type; R: Revenue; ROI: Return on Investment; TEI: Translational Efficiency Index; V –Translational Velocity (speed)

System-level performance can be summarized using either **bottleneck efficiency metrics** (minimum cycle velocity, min Vⱼ; Translational Efficiency Index–Bottleneck, TEI-B) or **average efficiency metrics** (weighted composite translational velocity, V-Mean; gain-based Translational Efficiency Index, TEI-G) (Table 1). Financial performance is assessed through global ROI and incremental efficiency (ΔV/ΔE).

In this framework, community transmission, as measured by the CTI, is evaluated by its capacity to accelerate translational velocities, increase innovation throughput, improve translational efficiency, enhance ROI, and reduce friction across cycles. Thus, CE is not assessed simply by participation volume but by its structural role in regulating translational speed, balancing performance across cycles, improving economic efficiency, and sustaining system impact.

The CE in a LHS can therefore be evaluated using the **CTI**, a structured multi-domain instrument (Table 2) designed to measure the extent to which community partners hold meaningful authority, accountability, and influence across governance, data, implementation, and equity processes. Projects are scored quarterly across eight domains (D1–D8) using a standardized 0–4 rubric. Each domain is independently evaluated by three raters - a member of the academic system, a health system representative, and a community evaluator - to ensure shared interpretation and credibility (target weighted Cohen’s κ ≥0.75). Domain scores include: shared governance, bidirectional accountability, data transparency and interpretability, participatory data governance, feedback closure and latency, co-production depth and integration, trust capital and partnership resilience, and equity integration. Scores are normalized, averaged and weighted to generate a composite CTI score (0–1, Table 2). This index reflects structural empowerment rather than participation volume, distinguishing transactional engagement from shared governance and fully community-anchored systems.

**Table 2:** Community transmission index (CTI) – formal instrument table including domains, scoring anchors, and data sources

CTI Domain (d)	Construct (what “transmission” means)	0 (Absent)	2 (Functional)	4 (Transformational)	Primary Evidence or Data Sources
**D1** Shared governance & decision rights	Community has formal authority, not advisory presence	No community roles in steering/policy	Standing advisory + occasional influence	Voting rights, co-chairs, defined decision domains	Committee rosters, bylaws, voting records
**D2** Bidirectional accountability	Clear obligations, enforcement, and reporting both ways	No accountability mechanisms	MOUs exist; limited enforcement	Shared accountability dashboards + corrective action pathways	MOUs, accountability scorecards, meeting minutes
**D3** Data transparency & interpretability	Community can see and understand performance	No community-facing metrics	Regular plain-language reports	Shared dashboards + co-defined measures	Dashboard access logs, dissemination materials
**D4** Participatory data governance	Community shares control over data rules/agenda	Data governance internal-only	Community reviews some metrics	Co-owned governance: access, use rules, analytic agenda	DUAs, governance charters, analytic agenda documents
**D5** Feedback closure & latency	Closed-loop learning with time targets	Feedback collected but not acted on	Some closures documented	Closed-loop standard + latency KPI (e.g., 30–60 days)	Issue trackers, timestamps: signal → change → confirmation
**D6** Co-production depth & integration	Community co-designs & co-implements with resource authority	Recruitment/dissemination only	Co-design OR co-implement	Co-lead/co-PI roles + budget share + authorship norms	Grant budgets, role descriptions, authorship lists
**D7** Trust capital & partnership resilience	Trust measured longitudinally and stable under stress	Not measured	Periodic trust surveys	Trust as KPI; high retention; resilient during disruptions	Validated trust scales, retention logs, network stability
**D8** Equity operating system	Equity embedded in targets, incentives, and governance	Equity rhetorical only	Disparities tracked	Equity tied to accountability/incentives + improving disparity slopes	Equity dashboards, QI metrics, incentive policies

Each project is scored quarterly across the eight domains (D1–D8) using a structured 0–4 rubric. Scores are assigned independently by three trained evaluators (one representative from the academic partner, one health system representative, and a community stakeholder), normalized, averaged, then weighted. Inter-rater reliability is assessed using weighted Cohen’s kappa (target ≥ 0.75). Major discrepancies are resolved via consensus review. Normalized domain scores are averaged. weighted and summed to produce a CTI value ranging from 0 to 1. KPI: key performance Indicator; MOU: memorandum of Understanding; QI: quality Improvement; A: Academia; H: health System; C: community

**Scoring Formula:** each domain scored ${r_{p,d}} \in \left( {0,1,2,3,4} \right)$, normalized ${s_{p,d}} = {r_{p,d}}/4$, then the three scores are averaged: ${S_{p,d}} = ({s_{A,p,d}} + {s_{H,p,d}} + {s_{C,p,d}}$**Composite:** ${\rm{CT}}{{\rm{I}}_p}\left( t \right) = \mathop \sum \limits_{d = 1}^8 {w_d}{S_{p,d}}\left( t \right)$, $\mathop \sum w_d= 1$.

For example, one can use a set of:

- default weights (balanced drivetrain): for w = **D1**: 0.16; **D2**: 0.10; **D3**: 0.12; **D4**: 0.10; **D5**: 0.16; **D6**: 0.12; **D7**: 0.12; **D8**: 0.12

- equity-forward weights: for w - increase **D8** to 0.20 and reduce **D2**, **D3**, and **D4** proportionally.

Range: 0–1; Interpretation (see also Fig. 5):

•[0–0.25) = Transactional

•[0.25–0.50) = Advisory

•[0.50–0.70) = Collaborative

•[0.70–0.85) = Shared Governance

•[0.85-1.00] = Community-Anchored LHS

Longitudinal tracking of CTI alongside system performance metrics (e.g., translational velocity, innovation throughput, return on investment, ROI), allows evaluators to determine whether empowerment is increasing, whether equity is institutionalized in decision-making, and whether community influence measurably shapes policy, implementation, and sustained impact. Table 3 illustrates how projects can be mapped to milestones across the four LHS cycles and how domain weights can be applied to compute CTI scores for evaluation purposes.

**Table 3:** Example of translational milestones mapped to the four LHS wheels, where milestone $k$ contributes differently to each learning cycle

Milestone (k)	Translational Stage	Clinical Care Wheel (j = 1)	Education Wheel (j = 2)	Research Wheel (j = 3)	Governance Wheel (j = 4)	Primary Data Source
**K**_**1**_	Community priority identification	0.20	0.15	0.25	**0.40**	Community forums, priority-setting records
**K**_**2**_	Study or intervention design finalized	0.10	0.25	**0.50**	0.15	Protocol approvals, curriculum committees
**K**_**3**_	Regulatory or IRB approval	0.05	0.05	**0.70**	0.20	IRB systems
**K**_**4**_	Pilot implementation launch	**0.45**	0.20	0.25	0.10	EHR or QI logs
**K**_**5**_	Pilot evaluation completed	0.35	**0.30**	0.25	0.10	Evaluation datasets
**K**_**6**_	Scale decision and policy alignment	0.25	0.15	0.20	**0.40**	Governance minutes, budget decisions
**K**_**7**_	Broad implementation	**0.50**	0.20	0.15	0.15	Adoption metrics
**K**_**8**_	Sustained integration (maintenance)	0.30	0.20	0.15	**0.35**	Longitudinal dashboards

Rows sum to 1 across wheels (interpretation weights). The conceptual insight is that early stages are often dominated by research and governance; mid-stages are typically dominated by clinical and education; while sustainment is strongly influenced by governance wheel. This explicitly formalizes why many translational efforts fail at scale: governance velocity is often the main bottleneck

**Fig. 1:**
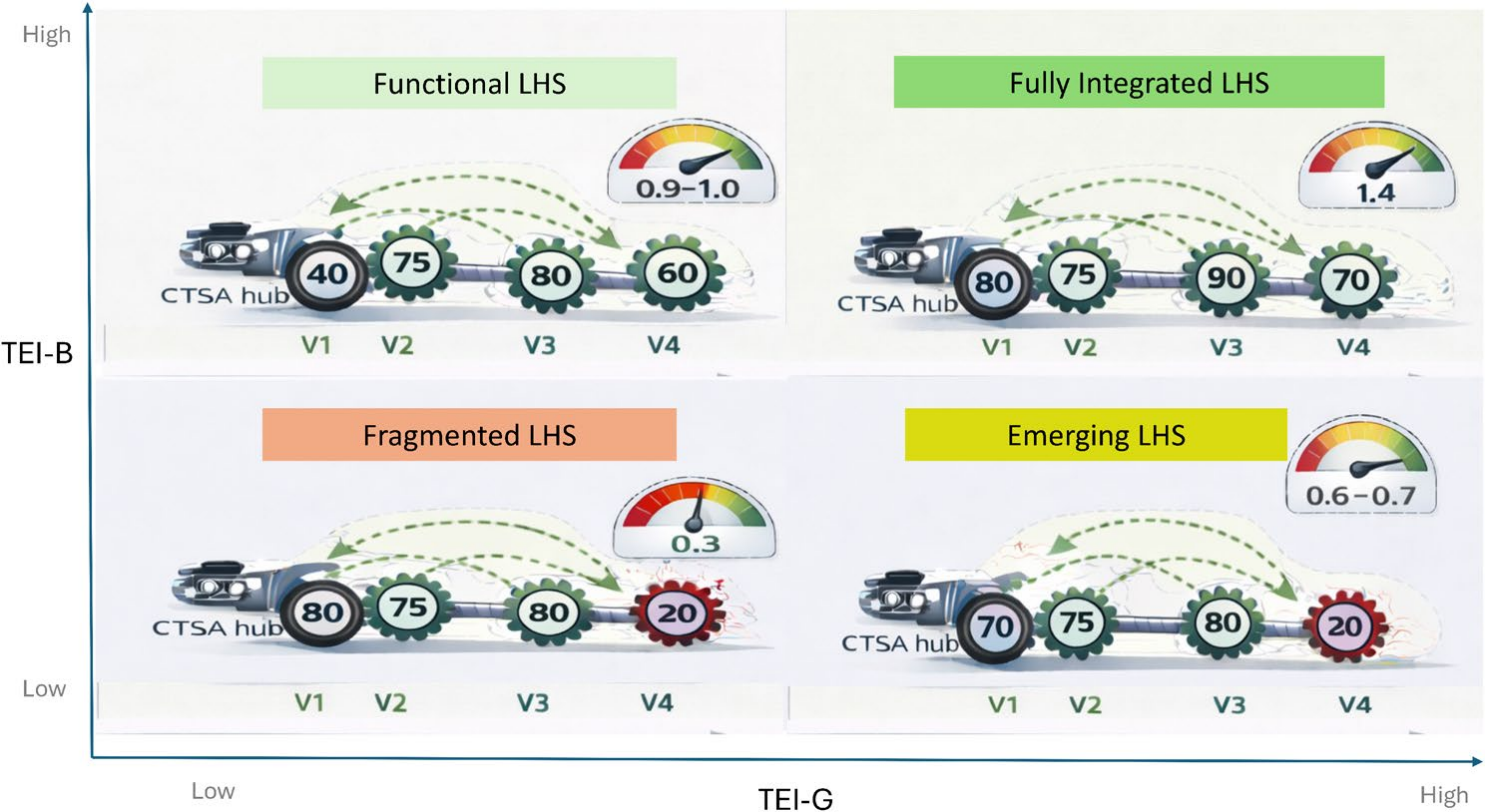
Translational efficiency typology of learning health systems (LHS). The diagram maps LHS performance across two efficiency dimensions: TEI-G (gain-based transmission efficiency index, x-axis), representing amplification from cross-cycle learning spillovers, and TEI-B (bottleneck-based transmission efficiency index, y-axis), representing constraint from the slowest learning cycle. The four quadrants depict system types ranging from *fragmented LHS* (where weak learning spillovers and governance bottlenecks limit translation); *Emerging LHS* - spillover-rich but constrained systems (high TEI-G, low TEI-B), where cross-cycle learning exists but a slow wheel restricts overall speed; *Functional LHS* - balanced but weakly coupled systems (low TEI-G, high TEI-B), i.e., where wheels move at similar speeds but lack amplification from learning spillovers; and *Fully integrated LHS* (high TEI-G, high TEI-B), where strong spillovers and balanced cycle velocities produce maximal translational acceleration. The figure visually illustrates how system performance depends both on learning amplification across cycles and on removal of drivetrain bottlenecks

Figure 1 illustrates an assessment of four types of LHS based on their performance, as measured by bottleneck and gained-based translational efficiencies.

### Computational simulations of the LHS engine–drivetrain model

#### Monte Carlo uncertainty simulation

To evaluate the robustness of the LHS engine–drivetrain model under uncertainty, we conducted a Monte Carlo simulation in which key system parameters were sampled across plausible ranges. Each iteration simulated 1,000 translational projects, and 500 iterations were performed in total.

At the start of each iteration, parameters governing community transmission dynamics, including the logistic slope $a$, midpoint ${m_0}$, maximum friction reduction ${r_{max}}$, and Hill parameters $\eta$ and $c$, were sampled from *uniform distributions* reflecting uncertainty in empirical values. Similarly, the cross-cycle spillover matrix $K$was scaled by a stochastic factor to represent variability in learning interactions among clinical care, education, research, and governance cycles. Stability of the coupled system was ensured by constraining the *spectral radius of* $K$* t*o values below one.

For each simulated project within an iteration, CTI values were drawn from *beta distributions* representing heterogeneous community engagement maturity. Engine power and friction parameters were sampled from *log-normal distributions*, reflecting variability in institutional resources and contextual barriers.

Wheel-specific translational velocities were computed using the drivetrain equations described above, followed by calculation of TEI-G, TEI-B, overall TEI, innovation throughput, and economic outputs. Iteration-level outcomes included: median TEI values, mean translational velocities, innovation counts, financial returns, and correlations between CTI, translational efficiency, and ROI. Additionally, each project was classified into one of four LHS states—fragmented, emerging, functional, or integrated—based on TEI thresholds. Across Monte Carlo iterations, summary statistics and 95% simulation intervals were computed to quantify the expected range of system behavior under parameter uncertainty.

This Monte Carlo approach allowed evaluation of the stability of the proposed evaluation model and demonstrated how CE, learning spillovers, and bottlenecks jointly influence translational efficiency and system performance within LHS.

#### Statistical analysis

All simulations and statistical analyses were conducted using Python (NumPy, pandas, and Matplotlib) and JMP 19.0 (SAS Institute, Cary, NC).

Translational performance metrics, including gain-based translational efficiency (TEI-G), bottleneck efficiency (TEI-B), overall translational efficiency (TEI), innovation throughput, and return on investment (ROI), were computed for each simulated project. Relationships among CTI, translational efficiency, and economic performance were evaluated using Pearson correlation coefficients and descriptive statistics.

For the Monte Carlo simulation, key model parameters governing community transmission, friction reduction, and cross-cycle learning spillovers were randomly sampled across predefined ranges for each iteration. Summary statistics across iterations were reported as means, standard deviations, and 95% simulation intervals (2.5th–97.5th percentiles) or medians whenever non-normal distributions were encountered. System-level states (fragmented, emerging, functional, or integrated LHS) were classified using predefined TEI thresholds. All simulations were **deterministic within each iteration but stochastic across iterations due to parameter sampling**, allowing assessment of the robustness of the proposed evaluation framework under uncertainty.

### Deterministic system simulation

To illustrate the operational behavior of the LHS-CTSA engine–drivetrain model evaluation framework, we constructed a computational simulation representing translational performance across the four coupled learning cycles, i.e., clinical care, education, research, and governance. Full definitions of the equations below and computational steps can be found in the Electronic Supplementary Material 1.

For each simulated project $p$, a CTI value ${M_p}$ was sampled from a beta distribution reflecting realistic variation in community engagement maturity. Community transmission influences translational performance through two functions. Transmission efficiency was modeled as a logistic function,

$$T\left( M \right) = {1 \over {1 + {\rm{exp}}\left[ { - a\left( {M - {m_0}} \right)} \right]}},$$

which represents the proportion of institutional capacity converted into effective translational effort. Community engagement also reduces organizational friction through

$$R\left( M \right) = {r_{max}}{{{M^\eta }} \over {{M^\eta } + {c^\eta }}},$$

which models reductions in mistrust, misalignment, and coordination barriers.

Wheel-specific direct translational drive for each LHS cycle $j$ was defined as

$${f_{p,j}}\left( M \right) = {v_{max,j}}{{{E_{p,j}}T\left( M \right)} \over {{E_{p,j}}T\left( M \right) + {F_{p,j}}\left( {1 - R\left( M \right)} \right)}},$$

where ${E_{p,j}}$ represents CTSA engine power allocated to cycle $j$, ${F_{p,j}}$ represents contextual friction, and ${v_{max,j}}$ denotes the maximum attainable velocity for that cycle.

Cross-cycle learning spillovers were represented using a coupling matrix $K$, describing how activity in one learning cycle accelerates progress in another. The resulting coupled system velocities were calculated as

$${{\bf{v}}_p}\left( t \right) = {(I - K)^{ - 1}}{{\bf{f}}_p}\left( t \right).$$

System performance was evaluated using the Translational Efficiency Index (TEI), which combines two complementary metrics. The gain-based efficiency index (TEI-G) quantifies amplification produced by cross-cycle learning:

$$TEI{\rm{ - }}G = {{\parallel {\bf{v}}{\parallel _W}} \over {\parallel {\bf{f}}{\parallel _W}}}.$$

The bottleneck-based efficiency index (TEI-B) measures the degree to which system performance is constrained by the slowest learning cycle:

$$TEI{\rm{ - }}B = {{\mathop {\min }\limits_j \left( {{v_j}} \right)} \over {\mathop \sum \nolimits_j {\alpha _j}{v_j}}}.$$

The global translational efficiency was defined as $TEI = TEI{\rm{ - }}G \times TEI{\rm{ - }}B$.

Innovation throughput was modeled as a linear function of cycle velocities, while economic outputs, including **revenues, costs, and return on investment (ROI)**, were calculated using parameterized revenue and cost functions associated with each learning cycle. The deterministic simulation generated **project-level outcomes for translational velocity, innovation production, and financial performance**, enabling visualization of the relationships among community engagement, translational efficiency, and economic return.

## Results

### General characteristics

In our simulations, using the proposed measurements and derived metrics, from all iterations (*n*-12500), each one had on average (95% Confidence Interval, CI) 25 (18–31) projects, with a standard deviation (SD) of 3 projects. Translational velocities of the four LHS cycles followed normal distributions, with mean (95% CI)/SD for v_1_ (clinical), v_2_ (education), v_3_ (research) and v_4_ (governance) of 0.753 (0.745–0.761)/0.09, 0.591 (0.584–0.597)/0.072, 0.729 (0.721–0.737)/0.089, and 0.601 (0.721–0.737)/0.082 (month^−1^). The system’s bottleneck and weighted average velocities (95% CI)/SD were 0.560 (0.418–0.694)/0.074, and 0.668 (0.503–0.816)/0.083, respectively. Across iterations, the mean CTI was 0.523 (0.51–0.589), with a SD of 0.037. The median/SD for TEI-B and TEI-G were 0.833 (0.805–0.857)/0.013, and 1.495 (1.392–1.613)/0.067, respectively. Their product, TEI’s median (95% CI)/SD was 1.245 (1.126–1.360)/0.068. The organization’s mean (95% CI)/SD expenditures were $1,409,088 ($1,308,151–$1,506,702)/$50,257, while revenues were $2,868,387 ($2,138,137-$3,594,741)/$372,799. The global Return On Investment (ROI) was 1.95 (1.47–2.36)/0.22 for these organizations.

### LHS typology and translational efficiency

The conceptual typology of LHS illustrated in the Fig. 1 (TEI-G vs TEI-B quadrant diagram) illustrates how translational performance emerges from the interaction between learning spillovers and drivetrain bottlenecks. As such, four distinct system states are observed:

- *Fragmented LHS* - systems in this quadrant exhibit both low spillover amplification (low TEI-G) and strong bottlenecks (low TEI-B). In the example configuration, governance velocity (v_4_) is markedly slower than the other cycles, creating a drivetrain constraint that prevents translation despite strong activity in clinical and research cycles.
- *Emerging LHS* - these systems demonstrate moderate learning spillovers but remain constrained by one slow cycle—often governance or operational leadership—limiting overall system acceleration.
- *Functional LHS* - in functional systems, the four learning cycles operate at relatively balanced velocities, resulting in high TEI-B (few bottlenecks). However, spillover amplification remains modest, so system translation improves but does not yet reach maximal acceleration.
- *Fully Integrated LHS* – the highest-performing systems combine strong cross-cycle spillovers with balanced cycle velocities. In these systems, the CTSA hub (engine) effectively converts institutional capacity into translational motion across all four cycles simultaneously.

Together, the typology suggests that translation is constrained not simply by resources but by the structure of the system drivetrain, particularly governance capacity and cross-cycle learning.

When divided by these a priori criteria into 4 types of LHS (using medians for TEI-B and TEI-G), <1% of the organizations fell into the Fragmented LHS category, while 2%, 37% and 61% fell into Emerging, Functional or Fully Integrated LHS categories, respectively. The median (IQR) CTI was 0.226 (0.144–0.322), 0.389 (0.198–0.603), and 0.852 (0.613–0.971) for Emerging, Functional or Fully Integrated LHS, respectively (*p* < 0.0001, non-parametric Kruskal-Wallis test). Lastly, the median/interquartile range (IQR) ROI for Emerging, Functional or Fully Integrated LHS were 0.717 (0.386–1.201), 1.683 (0.972–2.278), and 2.403 (1.887–2.660). respectively (*p* < 0.0001, non-parametric Kruskal-Wallis test).

### Cross-cycle learning spillovers

**Fig. 2:**
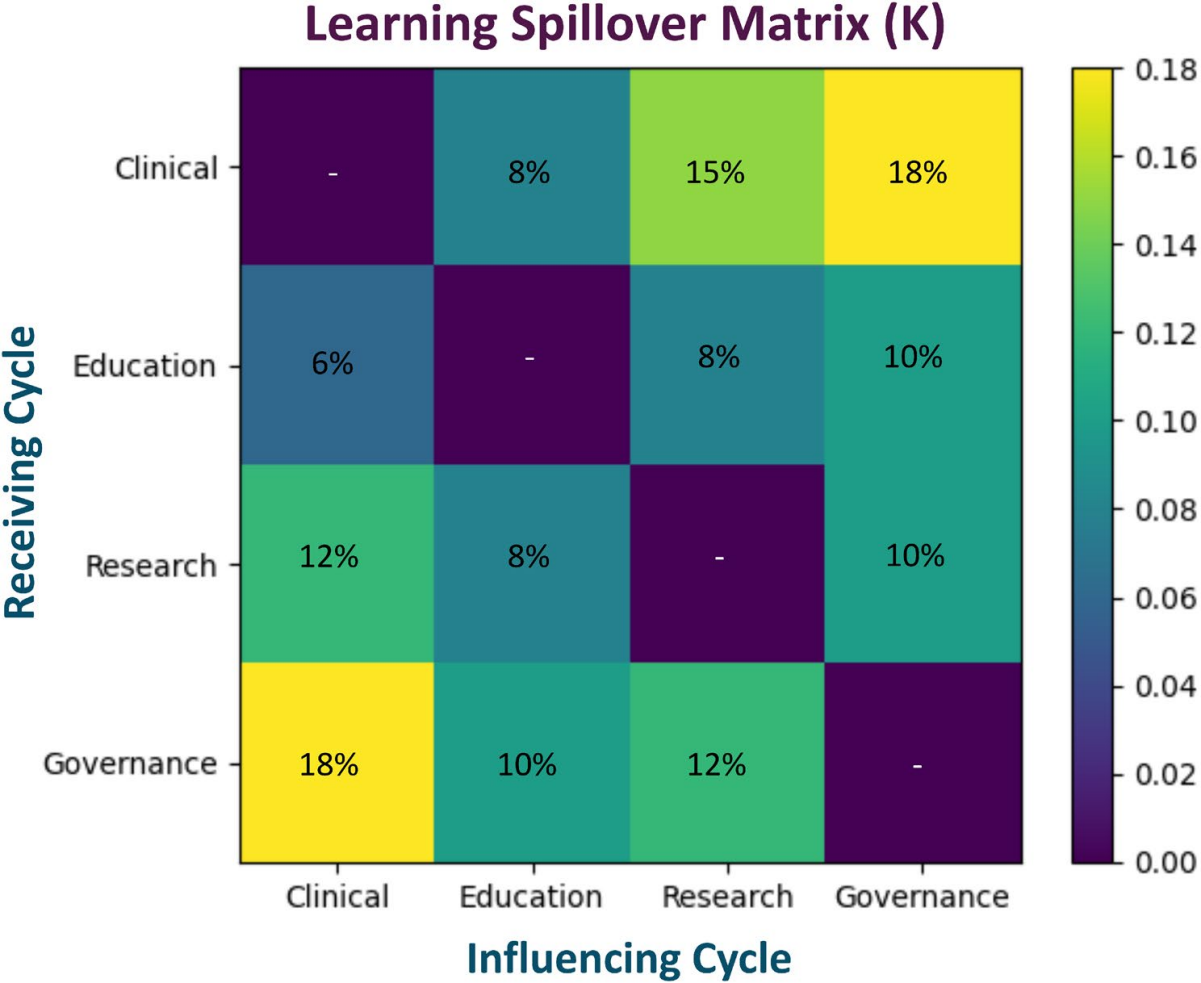
Learning spillover matrix in the LHS engine–drivetrain model. The matrix $K$ represents cross-cycle learning spillovers among the four learning health system cycles (clinical care, education, research, and governance). Cell values indicate the proportional acceleration of the receiving cycle (rows) generated by activity in the influencing cycle (columns). Diagonal entries are zero because cycles do not self-amplify within this representation. Color intensity reflects spillover magnitude, while numeric annotations indicate the percentage contribution. Larger values indicate stronger cross-domain learning effects that contribute to system-level translational acceleration

The learning spillover matrix (K) quantifies the degree to which progress in one cycle accelerates another (Fig. 2). Several patterns became apparent:

- Governance as a major system accelerator: governance exerts the strongest spillover influence on other cycles: Governance → Clinical: 18%; Governance → Research: 10%; Governance → Education: 10%. This finding reinforces the hypothesis that institutional alignment and leadership structures play a pivotal role in enabling translation across domains.
- Clinical innovation strongly feeds research: clinical care innovations generate substantial feedback to research (12%), consistent with the LHS paradigm in which clinical implementation produces new scientific questions and data streams.
- Education produces moderate but broad spillovers. Education generates modest but consistent spillovers across all cycles, suggesting that workforce development acts as a system stabilizer rather than a primary accelerator.

Overall, the matrix highlights that translation is not linear but networked, with governance and clinical cycles functioning as major hubs of learning diffusion.

### Translational velocities and economic outcomes

**Fig. 3:**
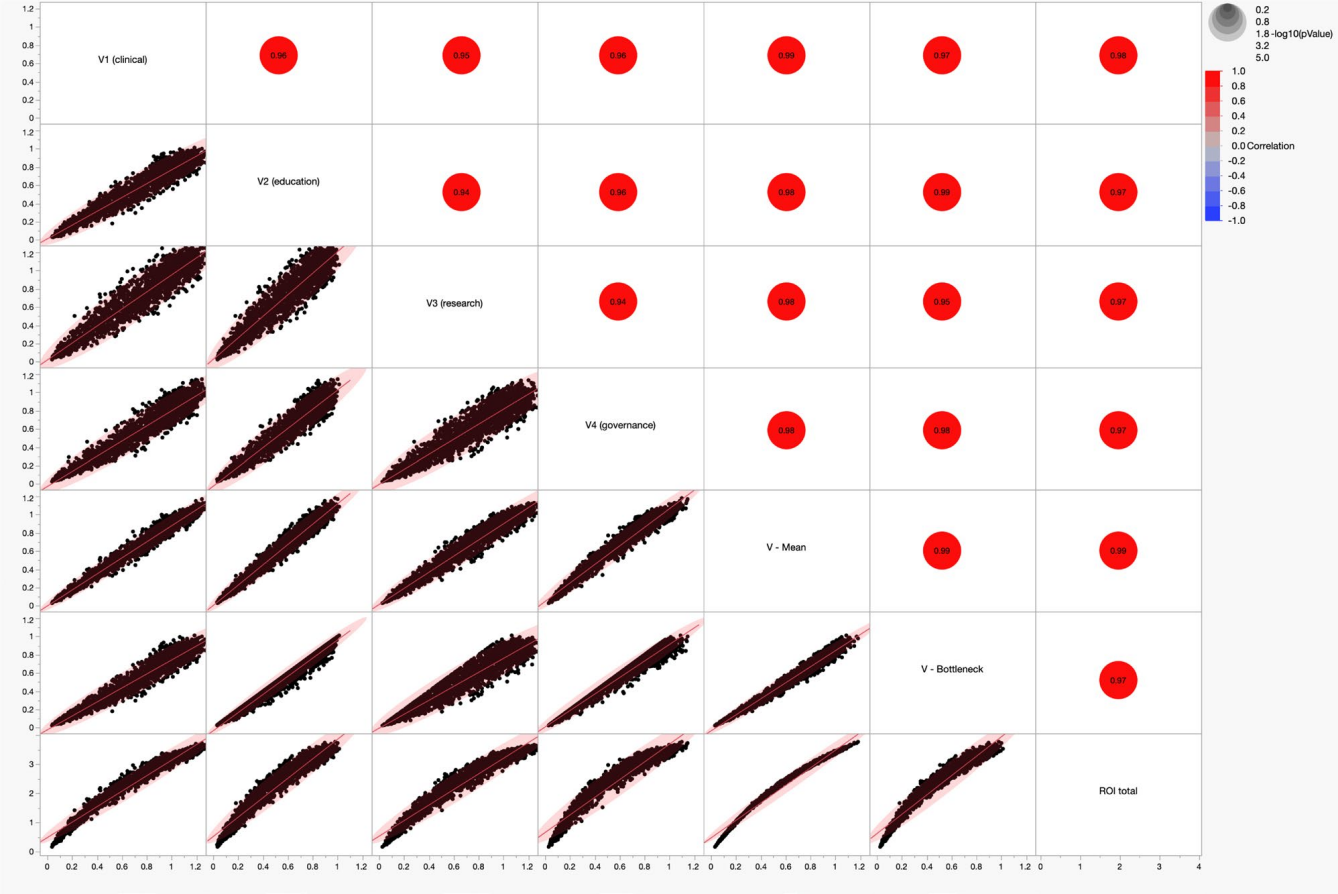
Multivariate analysis of global return on investment (ROI) vs different learning health system (LHS) cycle velocities. The graph illustrates high levels of reciprocal correlation - red dots show the specific r, correlation coefficients, while the lower triangle illustrates linear regression scatterplots with 95% confidence ellipses for the relationships between translational velocities v_1_ (clinical), v_2_ (education), v_3_ (research), v_4_ (governance), system bottleneck velocity (V-Bottleneck) and system weighted average velocity (V-Mean) vs the global, institutional ROI. The high degree of collinearity between v_1_, v_2_, v_3_ and v_4_ is concordant with the model’s observed learning spillovers

The multivariate analysis of ROI versus cycle velocities (Fig. 3) shows strong positive correlations between translational velocity and economic performance across all four cycles. Several important observations deserve mention:

- All four cycles contribute to economic value

The scatterplot matrix demonstrates strong correlations between ROI and each cycle velocity (V_1_–V_4_), indicating that translational performance is not driven solely by clinical implementation but by the combined functioning of the entire system.

- Governance velocity is particularly influential

Among the four cycles, governance velocity shows one of the strongest relationships with ROI. This supports the hypothesis that leadership alignment, policy agility, and institutional coordination reduce translational friction.

- System velocity metrics outperform individual cycles

Aggregate measures such as mean velocity and bottleneck velocity exhibit even stronger relationships with ROI than individual cycles. This suggests that system-level integration matters more than isolated excellence in one domain.

### Economic returns and translational cycle performance

**Fig. 4:**
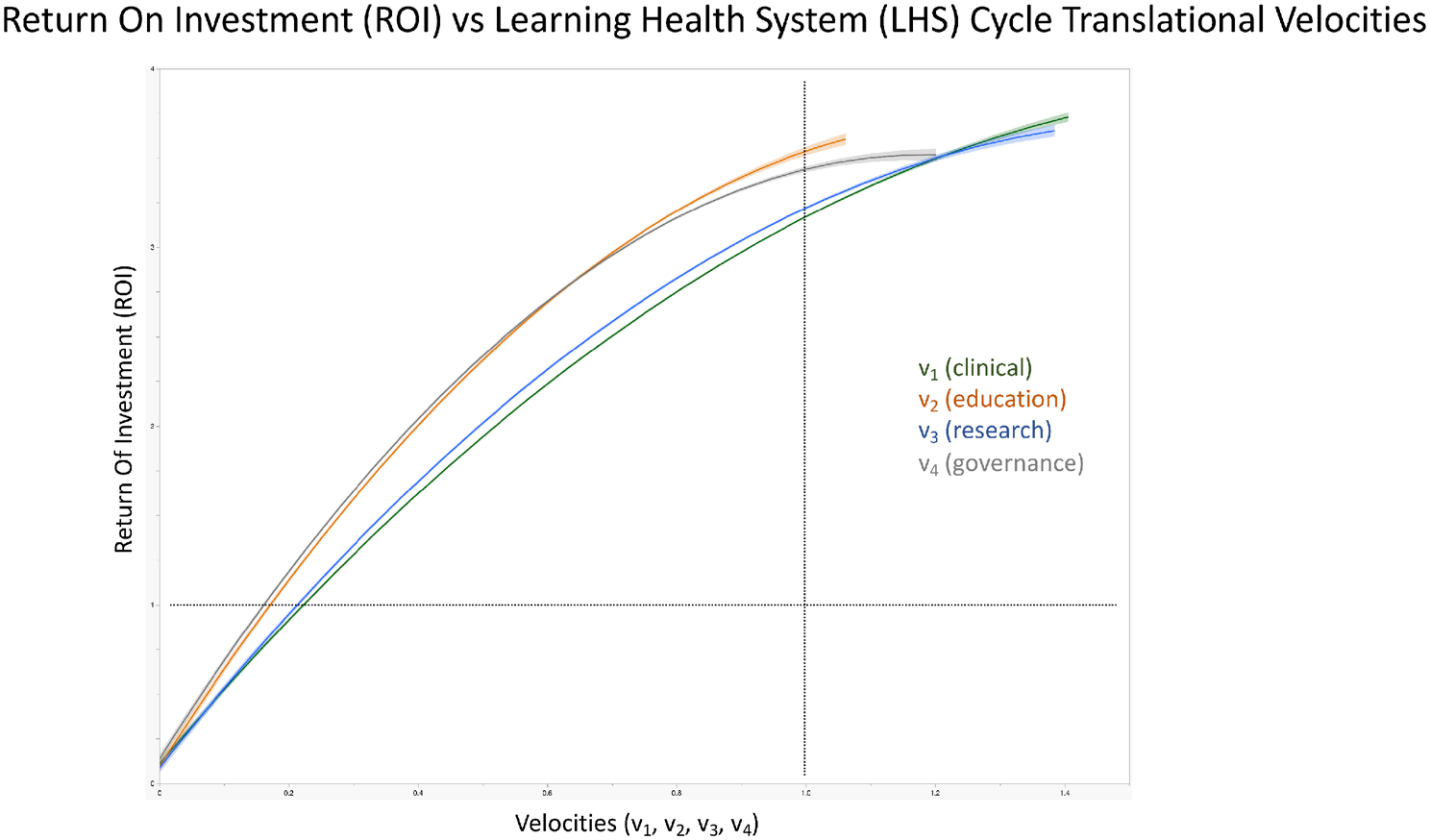
Return on investment (ROI) vs translational velocities. Global (institutional level) ROI plotted against different translational velocities of the LHS cycles or wheels. Velocity is the inverse of the project latency to sustainment (in months) – for example, a velocity of 1 corresponds to a latency of 1 month, a velocity of 0.5 = 1/2 a latency of 2 months, a velocity of 0.2 = 1/5 a latency of 5 months, etc. The simulation shows that for projects of 1 month duration until sustainment (the vertical dotted line), the ROI is the highest in the education and governance domains (3.5 and 3.4, respectively), while for clinical and research domains the ROI is closest to 3.1. Similarly, at the threshold of 1.0 for ROI (horizontal dotted line), education and governance-based successful projects ready for sustainment are of ~6.5 and ~5 months in duration for clinical and research projects, respectively. For educational and governance projects of 0.2 velocities (~5 months in duration), the ROI jumps up to ~1.4

The relationship between ROI and the individual cycle velocities further reinforces the central thesis of the engine–drivetrain model (Fig. 4). The figure demonstrates a generally monotonic relationship in which (1) increasing translational velocity leads to progressively higher economic returns; and (2) gains appear nonlinear, with ROI accelerating more rapidly once velocities exceed moderate thresholds. This suggests the presence of network effects in translational science, where improvements in one domain propagate through spillover mechanisms to amplify system productivity.

### Community transmission and system performance

**Fig. 5:**
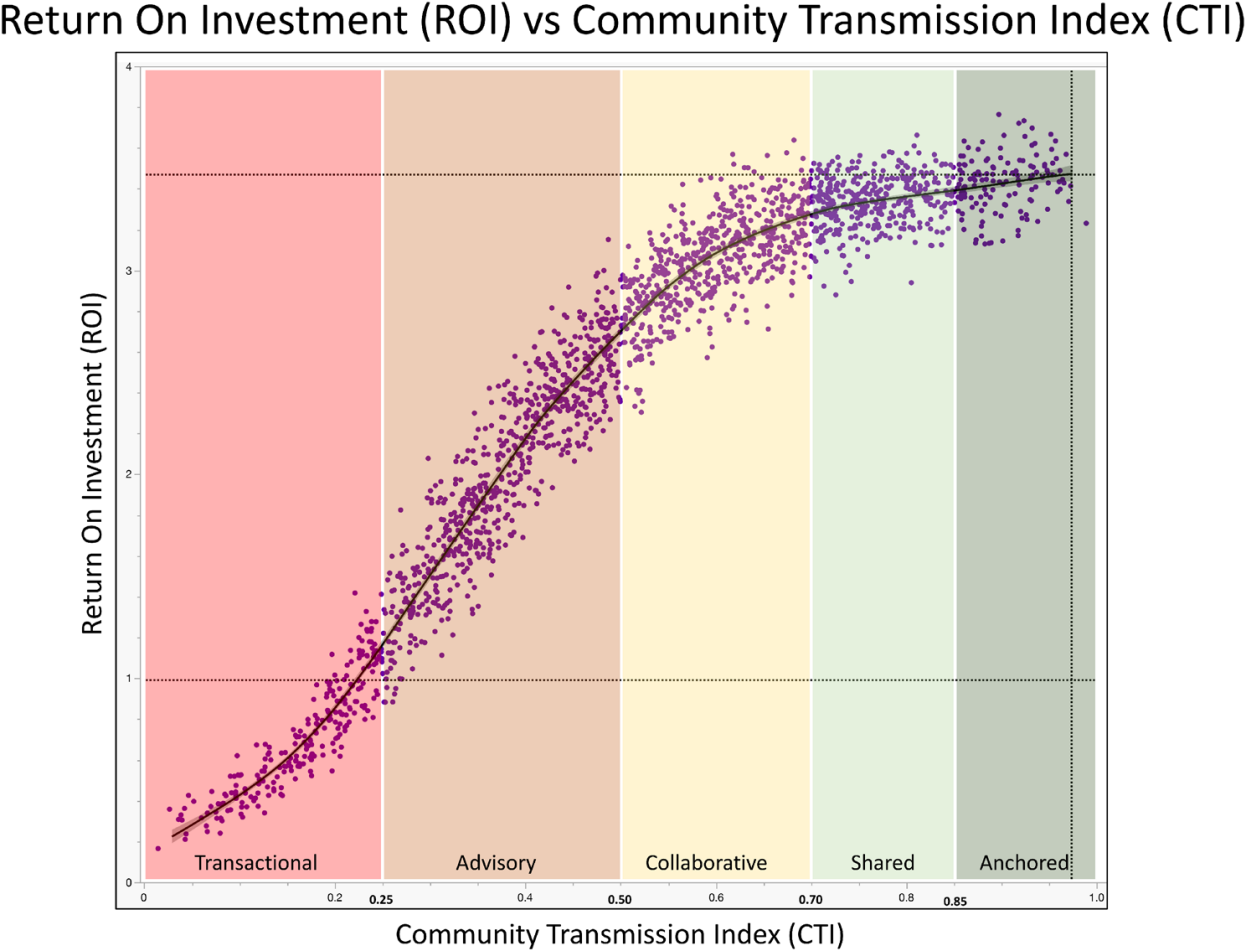
Relationship between community transmission index (CTI) and return on investment (ROI) in the LHS engine–drivetrain model. This graph illustrates the non-linear relationship between the maturity of community empowerment (CE) and the economic performance of translational initiatives within a learning health system (LHS). The x-axis represents the community transmission index (CTI), a composite score (0–1) reflecting the degree of CE across governance, data transparency, accountability, co-production, trust, and equity domains. The y-axis represents the global return on investment (ROI), derived from translational activities across the four LHS cycles (clinical care, education, research, and governance), and calculated as the ratio of system revenue generated to institutional expenditures. Each point represents a translational project. The upward trend illustrates that higher CTI values are associated with improved translational efficiency and greater economic returns, reflecting the role of CE as the transmission mechanism that converts CTSA hub resources into sustained system-level impact. In our simulations, the threshold of 1 for ROI is accomplished with a CTI of ~0.25 (lower dotted line, the transition between transactional and advisory types of relationships), while for projects with maximal ROI (~3.5), the CTI is ~0.97 (as community-anchored LHS)

The strongest and most striking relationship is observed between CTI and ROI (Fig. 5). The curve demonstrates a clear nonlinear increase in economic returns as CE matures. The relationship is sigmoidal, suggesting a threshold effect, i.e., once community engagement becomes structurally embedded in governance and decision-making, system performance accelerates rapidly. This supports the conceptualization of community as the transmission mechanism in the LHS drivetrain.

### Comparative system states

**Fig. 6:**
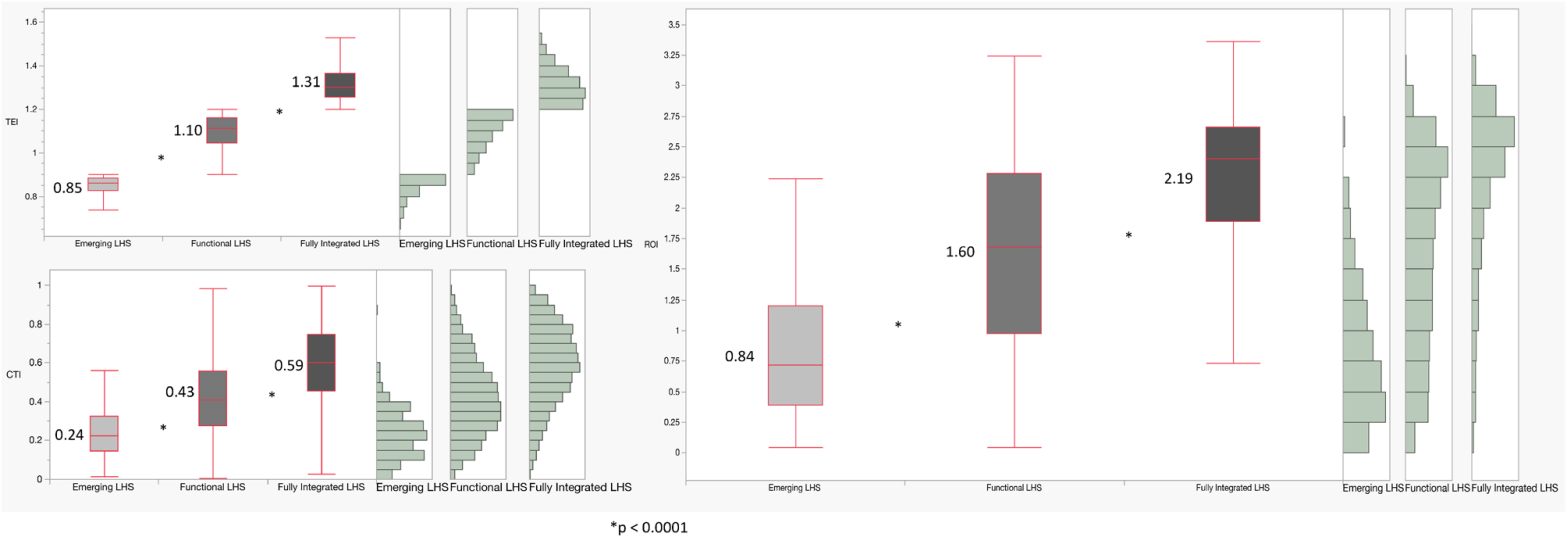
Main global performance assessment measurements for different learning health system types (Emerging, Functional, Fully Integrated; fragmented – excluded due to insufficient representation in the simulation model and likely un-necessary for design purposes). The graph represents the mean values for translation efficiency index (TEI), community transmission index (CTI) and return on investment (ROI); *p < 0.0001 by kruskall-Wallis non-parametric ranked sum tests

The final comparative analysis demonstrates how CTI, TEI, and ROI evolve across LHS maturity stages (Fig. 6). Median values increase systematically across system states, while the differences across stages are highly statistically significant (*p* < 0.0001). These results suggest that community engagement maturity and translational efficiency co-evolve, reinforcing each other as systems mature.

In a global multivariate model, global ROI can be estimated by Vmean, TEI and CTI as follows:

$$\begin{array}{l}{\rm{ROI}}\,{\rm{ = }}\,\,{\rm{0}}{\rm{.4587}}\,{\rm{ - }}\,{\rm{0}}{\rm{.20679 \bullet CTI}}\,{\rm{ - }}\,{\rm{0}}{\rm{.139885}}\, \bullet \\{\rm{TEI}}\,{\rm{ + }}\,{\rm{2}}{\rm{.650027 \bullet Vmean}}\end{array}$$

(adjusted R^2^ = 0.977, *p* < 0.0001, variance inflation factor < 7.3 for all predictive variables).

## Discussion

This study proposes and evaluates a conceptual and quantitative framework for understanding Learning Health System (LHS) performance using the engine–drivetrain model of translational science [1]. In this paradigm, the Clinical and Translational Science Award (CTSA) hub functions as the engine of translational capacity, the community serves as a stabilizing axle; community empowerment (CE) acts as the transmission mechanism; and four coupled learning cycles (clinical care, education, research, and governance) operate as the drivetrain wheels that convert institutional resources into sustained system impact. The results illustrate how translational efficiency and economic performance emerge from the interaction among these components.

The typology of LHS states defined by the interaction of gain-based translational efficiency (TEI-G) and bottleneck efficiency (TEI-B) highlights that system performance depends not only on the strength of learning spillovers but also on the absence of structural bottlenecks. Systems characterized by weak spillovers and strong constraints exhibit fragmented translation, whereas fully integrated systems combine strong cross-cycle learning with balanced cycle velocities (Fig. 4). This finding reinforces a core principle of complex adaptive systems: translational performance depends on networked learning interactions rather than isolated improvements within individual domains.

The learning spillover matrix (Fig. 2) further demonstrates that the four LHS cycles are strongly interconnected. Governance shows particularly strong spillover effects on other domains, suggesting that leadership alignment, policy agility, and institutional coordination may serve as critical accelerators of translational activity. Clinical innovation also exerts substantial feedback to research, consistent with the LHS concept that implementation generates new scientific questions and data streams. Education produces somewhat weaker but broadly distributed spillovers, indicating that workforce development acts primarily as a stabilizing infrastructure that sustains system learning.

Multivariate analyses (Fig. 3) show strong associations between translational velocities across the four cycles and return on investment (ROI). Notably, system-level metrics such as mean translational velocity and bottleneck velocity show slightly stronger relationships with ROI than individual cycle velocities. This suggests that integration across cycles produces greater economic value than isolated improvements in a single domain, supporting the systems-oriented design of CTSA hubs and similar translational infrastructures.

Another notable finding is the nonlinear relationship between CTI and ROI (Fig. 5). As community engagement matures from transactional all the way to community-anchored partnerships, translational efficiency and economic returns increase sharply. This pattern suggests the presence of threshold effects: once communities gain meaningful governance roles, transparent access to data, and co-production authority, the translational system begins to operate more efficiently. In this sense, community engagement acts as a mechanical transmission, converting the institutional power of the CTSA engine into effective system motion by reducing friction related to mistrust, misalignment, and inequity.

The comparative analysis of LHS states further demonstrates that improvements in CTI, TEI, and ROI occur in parallel as systems mature from emerging to fully integrated configurations (Fig. 6). These findings support the hypothesis that CE and translational efficiency co-evolve, reinforcing each other through iterative learning cycles.

Several implications arise from this work. First, the evaluative framework highlights governance as a potentially underappreciated determinant of translational performance, emphasizing the importance of leadership structures capable of aligning clinical, educational, and research missions. Second, the framework provides a quantitative basis for evaluating LHS maturity, using measurable system variables such as cycle velocities, learning spillovers, and community transmission. Finally, the model offers a practical tool for CTSA hubs and health systems to diagnose drivetrain bottlenecks and identify leverage points for accelerating translation.

This study has several limitations. The results presented here are derived from simulation-based modeling and conceptual parameterization rather than empirical data from a single health system. Although parameter ranges and distributions were selected to reflect plausible system behavior, future work should validate the framework using longitudinal data from CTSA hubs and other LHS initiatives. In addition, spillover effects and community transmission dynamics may vary across institutional contexts and should be empirically estimated in future studies.

The engine-drivetrain model provides a testable mechanism, i.e., that CTSA resources accelerate translation most effectively when community transmission maturity is sufficiently high to engage the learning system, close feedback loops, and reduce equity-related friction. The explicit inclusion of a governance wheel recognizes that scale and sustainability depend on policy and incentives. As CTI improves, the coupling matrix K strengthens, resulting in amplification of learning across all four cycles and improved resilience under system stress. In a four-wheel LHS, community engagement should therefore be conceptualized as a drivetrain property (an axle and variable transmission) rather than simple outreach activity.

Operationalizing this framework requires coordinated data collection across multiple domains. A highly functional LHS should put in place coordinated data collection across multiple domains, and a pragmatic, systematic evaluation based on CTI scoring (3-rater based scoring, as above); milestone timestamps across projects; wheel velocity dashboards, estimation of $\beta_{k}$ (stage sensitivity to CTI) and K (spillovers, cross-learning), comparative analyses of high-CTI vs low-CTI projects controlling for complexity, etc. Relevant data sources may include institutional dashboards, electronic health record analytics, research project tracking systems, governance meeting records, financial reports, CTSA translational workforce development, evaluation and QI cores, and community partnership surveys. Quarterly evaluation cycles allow organizations to monitor changes in system performance, in community engagement maturity and their interaction. Feedback from evaluations should be shared with institutional leaders and community partners alike to facilitate continuous learning and improvement. The evaluative framework is comprehensive, including measurements that are relevant to all constituents, i.e., members of the academic system (A), community (C), and leaders of the health system (H), such as TEI, CTI and ROI.

More broadly, this approach aligns with other translational evaluation frameworks, including the Translational Science Benefits Model, by capturing how the engine-transmission-axle machinery produces lasting health and societal benefits at local, national, and global levels [6, 7]. The national CTSA consortium further enables networked and distributive learning across institutions, allowing multiple translational engines to work in parallel while maintaining community-centered direction, and potentially adopting shared evaluation frameworks [8, 9].

## Conclusions

The engine–drivetrain model provides a systems-based concept for understanding how translational science infrastructures convert institutional resources into societal impact within LHS [1]. By explicitly modeling the role of community engagement as the transmission mechanism of the system, the model helps explain why organizations that embed communities in governance and co-production structures achieve higher translational efficiency and greater economic returns. This article may help guide future efforts to design, evaluate, and optimize LHS architectures across academic health systems.

This paper presents a conceptual and quantitative framework for evaluating LHS using the engine–drivetrain model of translational science, in which CTSA hubs function as the engine, community acts as a stabilizing axle, community engagement acts as the transmission, and four interconnected learning cycles (clinical care, education, research, and governance) serve as the drivetrain wheels. The analyses suggest that LHS performance depends not only on the speed of individual learning cycles but also on the strength of cross-cycle learning spillovers and the absence of structural bottlenecks. Higher levels of community engagement—captured through the Community Transmission Index (CTI)—are associated with improved translational efficiency and greater return on investment, underscoring the central role of community partnership in effective translation.

Together, these findings suggest that LHS achieve maximal impact when institutional capacity, governance alignment, and community empowerment operate as an integrated drivetrain system. The proposed framework offers a practical approach for evaluating, comparing, and optimizing translational science infrastructures within CTSA hubs and other complex health systems.

## Electronic supplementary material

Below is the link to the electronic supplementary material.

Supplementary material 1

## Data Availability

Not applicable

## Abbreviations

A: Academia
C: Community
H: Health(care) system
AI: Artificial Intelligence
ANN: Artificial Neural Networks
CE: Community Empowerment
CTI: Community Transmission Index
CTSA: Clinical and Translational Science Award;
LHS: Learning Health System
E: Expense (cost)
IOM: Institute of Medicine
IRB: Institutional Review Board
KPI: Key Performance Indicator
LLM: Large Language Models
MOU: Memorandum of Understanding
NCATS: National Center for Advancing Translational Science
NIH: National Institutes of Health
N5^th^ & N95^th^: 5^th^ & 95^th^ national percentiles of global LHS translational velocities
PDCA: Plan-Do-Check-Act cycle
PDCAd: PDCA for the data analysis phase
PDCAi: PDCA for the implementation phase
PDCAp: PDCA for the practice phase
P2D: Practice to Data
D2K: Data to Knowledge
K2P: Knowledge to Practice
QI: Quality Improvement
Qi5^th^ & Qi95^th^: 5^th^ & 95^th^ institutional percentiles of project speeds in LHS of i type
R: Revenue
ROI: Return on Investment
TS: Translational Science
UCLA: University of California Los Angeles
V –Translational: Velocity (speed)
