# Supplementary material for: Clinical and Translational Science Award hubs in learning health systems: evaluation framework of the engine-drivetrain model
Source: J Transl Med. 2026 Apr 22;24:737. doi: 10.1186/s12967-026-08123-z (PMC13237895; doi:10.1186/s12967-026-08123-z)
Supplement: Supplementary file 1 — Supplementary material 1 [file 12967_2026_8123_MOESM1_ESM.docx]

**Supplementary material:**

Definition of variables and the sequence of computational steps:

**1. Index:** variables and measurements:

1. $p$: project/initiative/intervention (QI bundle, implementation project, trial-to-practice pathway, curriculum change, etc);
2. $j=1,2,3,4$: LHS cycles or wheels (Clinical, Education, Research, Governance);
3. $k$: translational milestones (stage transitions);
4. $t$: time;
5. $v_{p}$: cycle velocities *(what we observe) -* each represents how fast that cycle learns/adapts;
6. $f_{p}:$ direct engine output, i.e., what each cycle would achieve *without cross-learning*. This depends on: CTSA resources (engine power)**;** CE maturity (transmission efficiency)**;** friction reduction. community affects $f_{p}$via efficiency increases or friction reduction;
7. M: transmission setting (0-1), represents the degree of CE maturity or CTI;
8. T(M): Transmission or gear efficiency (0-1), represents the fraction of engine power converted to motion;
9. exp(x): exponential function (used for smooth phase change, non-linear transitions).

**2. Core Quantities and Steps:**

1. Establish and order LHS **milestones** with timestamps:

$t_{p,j,0}\left( priority identification \right)<t_{p,j,1}<\cdots<t_{p,j,K}(sustained scale)$

Example: $k=0$ priority set (inaugural team meeting that decided the problem or intervention of interest) → $k=1$ protocol approved → $k=2$ pilot start → $k=3$ pilot complete → $k=4$ scale decision → $k=5$ sustained adoption.

1. Define **stage durations**:

$$\Delta t_{p,j,k}=t_{p,j, k+1}-t_{p,j,k}$$

2. Define *translational velocity* as **instantaneous stage velocity** and **overall translational velocity**:

$$v_{p,j,k}=\frac{1}{\Delta t_{p,j,k}},v_{p,j}=\frac{K}{t_{p,j,K}-t_{p,j,0}}$$

(or use “units of progress per month” if the milestones represent equal “progress units.”)

1. Create the **Translational Hazard Model** or Stage-based translational progress (milestones) linked to wheel velocities:

Define milestones $t_{p,0},\ldots,t_{p,K}$and stage durations $\Delta t_{p,k}$.

Let each milestone stage $k$“belong” primarily to one wheel $j(k)$(or have a mixture via weights).

Define a hazard of completing stage $k$:

$$\boldsymbol{h}_{\boldsymbol{p,k}}\boldsymbol{(t)=}\boldsymbol{h}_{\boldsymbol{0,k}}\boldsymbol{(t)}\mathbf{exp}\boldsymbol{}\left( \boldsymbol{\beta}_{\boldsymbol{k}}\text{ }\boldsymbol{M}_{\boldsymbol{p}}\boldsymbol{(t)+}\boldsymbol{\lambda}_{\boldsymbol{k}}^{\boldsymbol{\top}}\mathbf{v}_{\boldsymbol{p}}\boldsymbol{(t)+}\boldsymbol{\gamma}_{\boldsymbol{k}}^{\boldsymbol{\top}}\boldsymbol{X}_{\boldsymbol{p}}\boldsymbol{(t)} \right)$$

This comes from **survival analysis** or **time-to-event modeling**, adapted to translational science. Instead of modeling *death* or *failure*, we model **progress from one translational stage to the next**. The function is exponential because effects are multiplicative, not additive (CE scales system efficiency, it does not add fixed time savings).

Terms in the above equation:

- $h_{p,k}\left( t \right):$ **Translational Hazard (Speed of Progress $\boldsymbol{\propto}\text{Translational velocity}$),** conceptualized as the instantaneous probability that project $p$ completes translational stage $k$ at time $t$, given it has not yet completed it. Examples of stages: protocol finalized**;** first patient enrolled**;** pilot completed**;** implementation adopted**;** sustained scale reached**.** A higher $h_{p,k}(t)$ means faster progress.
- $h_{0,k}\left( t \right):$ **Baseline Translational Speed Engine** (or baseline Rotations Per Minute, RPM), which is the **default** speed of stage $k$ when CE is minimal and project characteristics are average**. This can be** represented by institutional bureaucracy**,** regulatory environment inertia, standard workflows, etc. In our model, this would be the engine idling without transmission engaged.
- $M_{p}\left( t \right):$**Community Transmission Index,** ${CTI}_{p}(t)\in[0,1]$. The CTI measures CE maturity (e.g., governance sharing, trust capital, feedback closure, co-production, equity integration, data co-ownership). Conceptually, CTI_p_ regulates how efficiently system energy becomes motion (*transmission efficiency*).
- $\beta_{k}:$**Transmission Gain Parameter** (or gear ratio sensitivity), which tells us how strongly CE maturity (CTI) accelerates stage $k$. If $\beta_{k}>0$ it means that engagement speeds translation; larger $\beta_{k}$means stronger effect. Example: $\beta_{k}=1.2$. Increasing CTI by 0.25 multiplies speed by: $e^{1.2\times0.25}\approx1.35$, ~35% faster stage completion. One key insight is that different stages have different sensitivities, as CE matters most downstream. For example, discovery phase has a small expected $\beta$, a trial set-up or activation may have a moderate $\beta,$ implementation typically will have large $\beta,$ while sustainability may have very large $\beta.$
- $\lambda_{k}$: coefficients denoting how wheel velocities accelerate that stage (e.g., governance wheel is key for scale/sustain decisions);
- $\lambda_{k}^{\top}\mathbf{v}_{p}\left( t \right):$ denotes a **weighted average** of the **wheel velocities** (λ_1_v_1_ + (λ_2_v_2_ + (λ_3_v_3_ +…);
- $X_{p}\left( t \right):$ **Project Context Variables** (road conditions), which are typically non-community factors such as funding size, intervention complexity**,** regulatory burden**,** clinical setting**,** workforce or organizational readiness, etc.
- $\gamma^{\top}\boldsymbol{X}_{p}\left( t \right):$ denotes a **weighted combination** of the project **context variables** (γ_1_X_1_+γ_2_X_2_+…), adjusting for confounders so the CTI effect is isolated.

In plain language, the above equation means that the speed at which a project moves through translational stages equals the baseline institutional speed multiplied by factors reflecting CE maturity and contextual conditions.

1. Compute **Community Transmission Index** (**CTSI**, a composite community empowerment or CE maturity score that operationalizes the transmission, see Table 2):

$$M_{p}(t)\in[0,1]$$

2. Wheel **velocities** (speed or velocity of learning/adaptation in each cycle or wheel):

$$v_{p}(t)=\left[ \begin{aligned} v_{p,1}(t) \\ v_{p,2}(t) \\ v_{p,3}(t) \\ v_{p,4}(t) \end{aligned} \right]$$

3. CTSA engine **power** for each wheel (represents resources and enabling capacity applied to each wheel, e.g., methods support, informatics, training infrastructure, governance facilitation, etc.):

$$E_{p}(t)=\left[ \begin{aligned} E_{p,1}(t) \\ E_{p,2}(t) \\ E_{p,3}(t) \\ E_{p,4}(t) \end{aligned} \right]$$

4. Contextual **friction** (represents sociotechnical resistance: workflow disruption, regulatory burden, mistrust legacy, inequity barriers, competing priorities, political or organizational constraints):
   $F_{p}(t)=\left[ \begin{aligned} F_{p,1}(t) \\ F_{p,2}(t) \\ F_{p,3}(t) \\ F_{p,4}(t) \end{aligned} \right]$
   1. Define the **Transmission Efficiency** **or Power Transfer Function** $\boldsymbol{T(M)}$ – equation:

$$T(M)=\frac{1}{1+\exp[-a(M-m_{0})]}\in(0,1)$$

Terms in the above equation:

- T(M): transmission or gear efficiency (0-1), fraction of engine power converted to motion;

- M: CTI, transmission setting (0-1), degree of CE maturity (a composite of shared governance, trust, co-production, feedback closure, equity integration); e.g., M=0: transactional outreach; M=1: community-anchored LHS;

- a: steepness parameter (>0), how rapidly efficiency improves (sensitivity to engagement); e.g., small (2-4) – gradual improvement; medium (6-10) clear maturity threshold; large (>12) – near phase transition (conceptually: low a – engagement improvements yield incremental gains; high a – system suddenly ‘clicks’ into learning mode);

- m_0_: engagement threshold or CTI level where acceleration begins (0-1) (gear engagement point); e.g., at M=m_0_, T(M)=0.5 (half of CTSA power becomes effective motion); typical expectation: m_0_ ≈ 0.40-0.60 (if m_0_ < 0.40, CE exists, but it does not change system behavior).

A logistic function like $T(M)$ is typically used when the system exhibits tipping points, phase transitions, and/or adoption thresholds, and is common in the diffusion of innovation, organizational change, epidemiology, or in neural activation models.

The function represents mathematically the fraction of the engine power transferred to the drivetrain (a logistic sigmoid function). The transmission efficiency describes how effectively CTSA resources (engine power) are converted into real-world translational motion as community empowerment (CE) maturity increases. Without CE maturity, institutional effort produces limited movement. With mature engagement, the same effort produces much larger change. This encodes a key hypothesis: LHS performance improves slowly at first, then accelerates rapidly once engagement becomes structural.

1. **Friction (Resistance) Reduction** **Function** $\boldsymbol{R(M)}$ – equation:

$$R(M)=r_{\max}\frac{M^{\eta}}{M^{\eta}+c^{\eta}}\in[0,r_{\max})$$

This is a Hill (saturation) function, widely used in biology and systems science. The Hill function like $R(M)$ is often used when early gains are small, when later gains accelerate, or saturation occurs, and is common in modeling enzyme kinetics, ecological adaptation, or learning curves. Friction reduction describes how CE maturity removes sociotechnical resistance that slows translation. Examples of friction: mistrust, workflow resistance, inequitable participation, policy barriers, misaligned incentives.

Terms in the above equation:

- R(M): friction reduction or lubrification effect (fraction of resistance removed, 0-r_max_);

- r_max_: maximum reducible friction (upper limit of friction removal as even perfect CE cannot eliminate all barriers, 0-1), structural ceiling; e.g., 0.40: institutional constraints dominate; 0.60-0.70: community strongly mitigates barriers; >0.80: highly adaptive system;

- η (eta): curvature parameter (how sharply friction falls, >0), non-linearity strength; controls early vs late friction removal; e.g., η = 1, gradual improvement; 2-3 threshold-like; >4: late rapid improvement; early engagement builds trust slowly; mature engagement rapidly removes barriers;

- c (friction reduction mid-point): half-saturation constant (CTI level for half friction reduction, 0-1), resistance breakpoint; e.g., when: $M=c$ , then $R(M)=\frac{r_{\max}}{2}$, meaning that half of the removable resistance disappears. Usually: $c\approx0.5$.

**How to Interpret Values Empirically**

Example parameters: $a=8,m_{0}=0.5,r_{\max}=0.65,c=0.55,\eta=2$

| CTI $M$ | Transmission $T(M)$ | Friction removed $R(M)$ | Meaning |
| --- | --- | --- | --- |
| 0.2 | 0.12 | 0.06 | transactional LHS |
| 0.4 | 0.31 | 0.22 | advisory LHS |
| 0.6 | 0.69 | 0.45 | collaborative LHS |
| 0.8 | 0.90 | 0.58 | Shared-governance LHS |
| 1.0 | 0.97 | 0.65 | community-anchored LHS |

As CE maturity increases, translational systems simultaneously improve the efficiency with which CTSA resources are converted into learning (transmission efficiency $T(M)$) and reduce sociotechnical resistance to change (friction reduction $R(M)$), producing nonlinear acceleration of LHS performance.

1. **Mathematical Model (4-wheel LHS):**

- **Direct wheel drive (uncoupled velocities):**

For each wheel $j$, define a “direct drive” velocity contribution:

$$f_{p,j}(t)=v_{\max,j}\text{ }\frac{E_{p,j}(t)\text{ }T(M_{p}(t))}{E_{p,j}(t)\text{ }T(M_{p}(t))+F_{p,j}(t)\text{ }[1-R(M_{p}(t))]}$$

where:

- $E_{j}\geq0$: CTSA “engine power” applied to wheel $j$(resources, infrastructure, enabling support)
- $F_{j}\geq0$: contextual friction for wheel $j$(sociotechnical resistance)
- $v_{\max,j}>0$: wheel scaling constant (max attainable velocity in your chosen units)
- $T(M)\in(0,1)$: transmission efficiency (power transfer)
- $R(M)\in[0,r_{\max})$: friction reduction from community maturity

Stack these into a vector:

$$\mathbf{f}_{p}\left( t \right)=\left[ \begin{aligned} f_{p,1}\left( t \right) \\ f_{p,2}\left( t \right) \\ f_{p,3}\left( t \right) \\ f_{p,4}\left( t \right) \end{aligned} \right]$$

This is what each wheel would achieve if it didn’t learn from the others.

- **Four-wheel coupling (community axle + LHS spillovers)**

Now define a **4×4 coupling matrix** $\boldsymbol{K}$ **(axle effect)** encoding learning spillovers:

$$K=\left[ \begin{matrix} 0 & \kappa_{12} & \kappa_{13} & \kappa_{14} \\ \kappa_{21} & 0 & \kappa_{23} & \kappa_{24} \\ \kappa_{31} & \kappa_{32} & 0 & \kappa_{34} \\ \kappa_{41} & \kappa_{42} & \kappa_{43} & 0 \end{matrix} \right]$$

Each element means: $\kappa_{ij}=\text{how much cycle j accelerates cycle i}$

Examples: research discoveries speed clinical change; clinical experience generates research questions; education improves implementation capability.

**Why is Community the axle?** Because spillovers require trust, shared meaning**,** legitimacy across domains**,** and without community the coupling is weak.

Meaning:

- $\kappa_{13}$: Research → Clinical spillover (evidence-based practice)
- $\kappa_{31}$: Clinical → Research spillover (practice-based research)
- $\kappa_{24}$: Governance → Education spillover (policy/incentives shaping curricula)
- $\kappa_{41}$: Clinical → Governance spillover (front-line signals shaping policy)
- **Extending the model to an entire LHS** (not a single project stage) **-** the coupled LHS wheel velocities are:

$$\mathbf{v}_{\boldsymbol{p}}\boldsymbol{(t)=(I-K}\boldsymbol{)}^{\boldsymbol{-1}}\mathbf{f}_{\boldsymbol{p}}\boldsymbol{(t)}$$

This is the machinery’s *drivetrain*: the axle enables coupling and the transmission modulates how much power enters the system; the matrix inverse captures recursive learning cycles.

Terms in the above equation:

- **I**: **4x4 Identity Matrix**, representing completely independent cycles (no interactions).
- $\boldsymbol{(I-K}\boldsymbol{)}^{\boldsymbol{-1}}$: **Amplification Operator** (reinforcing drivetrain)**,** key systems insight, encapsulating mathematically **recursive learning**, e.g., Clinical → Education → Research → Clinical → … or P2D → D2K→ K2P →P2D… Each loop adds acceleration. As such, $\left( I - K)^{-1} \right.$ captures **continuous system learning**. K represents the axle coupling between wheels. (I−K)^−1^=I+K+K^2^+K^3^+…
- $\boldsymbol{v}_{\boldsymbol{p}}\boldsymbol{:}$ Vehicle speed.

In a practical, numerical example, let’s assume that the baseline average time from pilot to sustained scale = 36 months. Estimated hazard model coefficient: $\beta=1.1$

***Case A:*** *Low CE maturity:* CTI = 0.30

Hazard multiplier:

$$\exp(1.1\times0.30)=\exp(0.33)=1.39$$

***Case B:*** *High CE maturity:* CTI = 0.75

Hazard multiplier:

$$\exp(1.1\times0.75)=\exp(0.825)=2.28$$

Relative hazard ratio (High vs Low):

$$\frac{2.28}{1.39}\approx1.64$$

**Interpretation:**

High-CTI projects complete stages ~64% faster. If baseline median time-to-scale = 36 months, then the predicted median under high CTI is:

$$\frac{36}{1.64}\approx22\text{ months}$$

This means that increasing CE maturity from advisory to shared governance levels could reduce time-to-scale from 36 months to ~22 months.

1. **Translational Efficiency Index (TEI)**

- **TEI-G: Gain-based Translational Efficiency Index** – Definition:

$$\boldsymbol{TEI}\text{-}\boldsymbol{G}_{\boldsymbol{p}}\boldsymbol{(t)=}\frac{\boldsymbol{\mid\mid}\mathbf{v}_{\boldsymbol{p}}\boldsymbol{(t)}\boldsymbol{\mid}\boldsymbol{\mid}_{\boldsymbol{W}}}{\boldsymbol{\mid\mid}\mathbf{f}_{\boldsymbol{p}}\boldsymbol{(t)}\boldsymbol{\mid}\boldsymbol{\mid}_{\boldsymbol{W}}}\boldsymbol{=}\frac{\boldsymbol{\mid\mid}\boldsymbol{(I-K}\boldsymbol{)}^{\boldsymbol{-1}}\mathbf{f}_{\boldsymbol{p}}\boldsymbol{(t)}\boldsymbol{\mid}\boldsymbol{\mid}_{\boldsymbol{W}}}{\boldsymbol{\mid\mid}\mathbf{f}_{\boldsymbol{p}}\boldsymbol{(t)}\boldsymbol{\mid}\boldsymbol{\mid}_{\boldsymbol{W}}}$$

TEI-G measures how much faster the entire LHS system moves compared with the direct effort applied to it (in engineering terms: gain = output magnitude/input magnitude)

The symbol:

$$\mid\mid x\mid\mid_{W}$$

is a weighted magnitude of a vector:

$$\mid\mid x\mid\mid_{W}=\sqrt{x^{\top}Wx}$$

Where:

$$W=\text{diag}(w_{1},w_{2},w_{3},w_{4})$$

The weights represent *mission emphasis* coefficients.

Example: let’s assume that the clinical, education, research and governance wheels receive the weights 0.35, 0.20, 0.25 and 0.20, respectively, depending on the institution’s priorities at the moment t. In this case, the weighted magnitude of the vector

$$v_{p}=\left[ \begin{aligned} v_{1} \\ v_{2} \\ v_{3} \\ v_{4} \end{aligned} \right]$$

is:

$$\mid\mid x\mid\mid_{W}=\sqrt{0.35{v_{1}}^{2}+0.20{v_{2}}^{2}+0.25{v_{3}}^{2}+0.20{v_{4}}^{2}}$$

Interpretation of the index: TEI-G = 1 - no amplification from learning cycles; TEI-G > 1 - cross-cycle learning accelerates translation; TEI-G > 2 - highly integrated LHS**.**

In other words, TEI-G measures how effectively a system converts learning spillovers into translational speed. If learning cycles are independent, v = f (TEI-G = 1). In a true LHS, research informs clinical caer, clinical experience generates new knowledge and leads to more research, governance enable scaling, and education may lead to training of implementers, etc. In this situation, spillover increases system velocity (v > f), which yields TEI-G > 1.

Because projects vary greatly, one may compute a robust hub metric using the median in order to reduce influence of more extreme projects (outliers):

**TEI-G_hub_ = median_p,t_(TEI-G_p_(t))**

- **TEI-B: Bottleneck-based Translational Efficiency Index** – Definition:

$\mathbf{TEI}\text{-}\mathbf{B}_{\mathbf{p}}\mathbf{(t)=}\frac{\mathbf{v}_{\mathbf{p,min}}\mathbf{(t)}}{\sum_{\mathbf{j=1}}^{\mathbf{4}} \boldsymbol{\alpha}_{\mathbf{j}}\mathbf{v}_{\mathbf{p,j}}\mathbf{(t)}}$

Numerator is represented by the bottleneck velocity, identifying the *slowest learning cycle:*

$$\mathbf{v}_{\mathbf{p,min}}\mathbf{(t)=}\underset{\boldsymbol{j\in(1,2,3,4)}}{\mathbf{min}}\mathbf{v}_{\mathbf{p,j}}\mathbf{(t)}$$

Denominator is the *weighted mean velocity* of the system.

$$\sum\alpha_{j}=1$$

Interpretation: TEI-B = 1 – wheels are balanced; TEI-B = 0.5 – mild bottleneck; TEI-B = 0.1 – severe bottleneck; TEI-B →0 – very slow governance processes.

The drivetrain analogy explains this very well: even if three wheels are spinning very quickly, a slow wheel limits forward motion.

- **Global** **TEI = TEI-G • TEI-B** (the product of amplification from learning spillovers and system balance)

TEI <0.5 indicates a fragmented system, 0.5-0.9 a partial LHS, 0.9-1.1 a functional LHS and >1.2 a highly integrated LHS.

A high performing LHS must have a strong cross-cycle learning and no dominant bottlenecks.

In summary, the TEI-G quantifies how much faster a LHS moves due to cross-cycle learning spillovers, while TEI-B measures the degree to which the slowest cycle constrains overall translational progress.

These indices are important because they connect systems engineering concepts with organizational learning, implementation science and translational science performance assessments, all within one single framework.

Running a 2,000-pass simulation (python code below), we obtained the following results for the above measures:

Overall summary ---

| **Metric** | **CTI** | **TEI-G** | **TEI-B** | **TEI** | **V-Bottleneck** | **ROI Total** |
| --- | --- | --- | --- | --- | --- | --- |
| Count (n) | 2000 | 2000 | 2000 | 2000 | 2000 | 2000 |
| Mean | 0.523 | 1.489 | 0.824 | 1.229 | 0.560 | 1.948 |
| Standard Deviation | 0.223 | 0.031 | 0.072 | 0.125 | 0.275 | 0.78 |
| Min | 0.026 | 1.328 | 0.45 | 0.657 | 0.03 | 0.162 |
| 25^th^ | 0.35 | 1.476 | 0.785 | 1.161 | 0.312 | 1.359 |
| 50^th^ (Median) | 0.537 | 1.498 | 0.835 | 1.251 | 0.635 | 2.248 |
| 75^th^ | 0.7 | 1.512 | 0.874 | 1.318 | 0.799 | 2.596 |
| Max | 0.995 | 1.532 | 0.98 | 1.488 | 1.057 | 2.999 |

Simulation model code:

from __future__ import annotations

import numpy as np

import pandas as pd

# ============================================================

# 1) Core LHS engine-drivetrain functions

# ============================================================

def transmission_efficiency(M: np.ndarray, a: float = 8.0, m0: float = 0.5) -> np.ndarray:

"""

T(M) = 1 / (1 + exp[-a(M - m0)])

Community transmission efficiency.

"""

M = np.clip(M, 0.0, 1.0)

return 1.0 / (1.0 + np.exp(-a * (M - m0)))

def friction_reduction(

M: np.ndarray,

r_max: float = 0.65,

eta: float = 2.0,

c: float = 0.55,

) -> np.ndarray:

"""

R(M) = r_max * M^eta / (M^eta + c^eta)

Fraction of reducible friction removed by community maturity.

"""

M = np.clip(M, 0.0, 1.0)

return r_max * (M**eta) / (M**eta + c**eta)

def direct_drive(

M: np.ndarray,

E: np.ndarray,

F: np.ndarray,

vmax: np.ndarray,

a: float = 8.0,

m0: float = 0.5,

r_max: float = 0.65,

eta: float = 2.0,

c: float = 0.55,

) -> np.ndarray:

"""

Wheel-specific direct drive:

f_j(M) = vmax_j * [E_j T(M)] / [E_j T(M) + F_j (1 - R(M))]

Inputs:

M: shape (n,)

E, F, vmax: shape (n, 4)

Returns:

f: shape (n, 4)

"""

T = transmission_efficiency(M, a=a, m0=m0)[:, None]

R = friction_reduction(M, r_max=r_max, eta=eta, c=c)[:, None]

num = E * T

den = num + F * (1.0 - R)

return vmax * num / np.maximum(den, 1e-12)

def coupled_velocity(f: np.ndarray, K: np.ndarray) -> np.ndarray:

"""

v = (I - K)^(-1) f

f: shape (n, 4)

K: shape (4, 4)

"""

I = np.eye(4)

A = np.linalg.inv(I - K)

return f @ A.T

# ============================================================

# 2) TEI metrics

# ============================================================

def weighted_norm_rows(X: np.ndarray, weights: np.ndarray) -> np.ndarray:

"""

||x||_W = sqrt(x^T W x), row-wise for X.

weights: length 4 vector

"""

return np.sqrt(np.sum((X**2) * weights[None, :], axis=1))

def tei_gain(v: np.ndarray, f: np.ndarray, w: np.ndarray) -> np.ndarray:

"""

TEI-G = ||v||_W / ||f||_W

"""

return weighted_norm_rows(v, w) / np.maximum(weighted_norm_rows(f, w), 1e-12)

def tei_bottleneck(v: np.ndarray, alpha: np.ndarray) -> np.ndarray:

"""

TEI-B = min_j(v_j) / sum_j alpha_j v_j

"""

v_min = np.min(v, axis=1)

v_mean = np.maximum(v @ alpha, 1e-12)

return v_min / v_mean

def tei_total(v: np.ndarray, f: np.ndarray, w: np.ndarray, alpha: np.ndarray) -> tuple[np.ndarray, np.ndarray, np.ndarray]:

"""

Returns TEI-G, TEI-B, TEI = TEI-G * TEI-B

"""

g = tei_gain(v, f, w)

b = tei_bottleneck(v, alpha)

return g, b, g * b

# ============================================================

# 3) Innovation, finance, and ROI

# ============================================================

def innovations_per_year(v: np.ndarray, innovation_scale: np.ndarray) -> np.ndarray:

"""

Simple innovation production function:

N_j = innovation_scale_j * v_j

"""

return v * innovation_scale[None, :]

def revenue_function(v: np.ndarray, N: np.ndarray, rev_v: np.ndarray, rev_n: np.ndarray) -> np.ndarray:

"""

R_j = rev_v_j * v_j + rev_n_j * N_j

"""

return rev_v[None, :] * v + rev_n[None, :] * N

def expense_function(

v: np.ndarray,

base_cost: np.ndarray,

pdca_p: np.ndarray,

pdca_d: np.ndarray,

pdca_i: np.ndarray,

cost_v: np.ndarray,

) -> np.ndarray:

"""

E_j = base_cost_j + cost_v_j * v_j + PDCA planning/development/implementation costs

"""

return (

base_cost[None, :]

+ cost_v[None, :] * v

+ pdca_p[None, :]

+ pdca_d[None, :]

+ pdca_i[None, :]

)

def roi(revenue: np.ndarray, expense: np.ndarray) -> np.ndarray:

"""

ROI_j = R_j / E_j

"""

return revenue / np.maximum(expense, 1e-12)

# ============================================================

# 4) Simulation setup

# ============================================================

def simulate_lhs_drivetrain(

n_projects: int = 1000,

seed: int = 42,

) -> pd.DataFrame:

rng = np.random.default_rng(seed)

# --------------------------------------------------------

# Community maturity (CTI)

# --------------------------------------------------------

# Beta distribution gives realistic spread in [0, 1]

M = rng.beta(2.2, 2.0, size=n_projects)

# --------------------------------------------------------

# Engine power E by wheel

# Order: [Clinical, Education, Research, Governance]

# --------------------------------------------------------

E = np.column_stack([

rng.lognormal(mean=0.20, sigma=0.25, size=n_projects), # clinical

rng.lognormal(mean=0.00, sigma=0.20, size=n_projects), # education

rng.lognormal(mean=0.30, sigma=0.30, size=n_projects), # research

rng.lognormal(mean=-0.05, sigma=0.20, size=n_projects), # governance

])

# --------------------------------------------------------

# Friction F by wheel

# Governance often carries larger friction

# --------------------------------------------------------

F = np.column_stack([

rng.lognormal(mean=np.log(0.8), sigma=0.25, size=n_projects),

rng.lognormal(mean=np.log(0.7), sigma=0.20, size=n_projects),

rng.lognormal(mean=np.log(0.9), sigma=0.30, size=n_projects),

rng.lognormal(mean=np.log(1.1), sigma=0.35, size=n_projects),

])

# --------------------------------------------------------

# Maximum attainable wheel velocities

# --------------------------------------------------------

vmax = np.tile(np.array([1.0, 0.9, 1.1, 0.85]), (n_projects, 1))

# --------------------------------------------------------

# Coupling matrix K

# Spillovers across the four wheels

# --------------------------------------------------------

K = np.array([

[0.00, 0.08, 0.15, 0.18], # Clinical receives from education, research, governance

[0.06, 0.00, 0.08, 0.10], # Education receives from clinical, research, governance

[0.12, 0.08, 0.00, 0.10], # Research receives from clinical, education, governance

[0.18, 0.10, 0.12, 0.00], # Governance receives from all others

])

# --------------------------------------------------------

# Compute direct drive and coupled velocities

# --------------------------------------------------------

f = direct_drive(M, E, F, vmax)

v = coupled_velocity(f, K)

# --------------------------------------------------------

# TEI weights

# --------------------------------------------------------

w = np.array([0.30, 0.20, 0.25, 0.25]) # mission emphasis

alpha = np.array([0.25, 0.25, 0.25, 0.25]) # balanced mean

tei_g, tei_b, tei = tei_total(v, f, w, alpha)

# --------------------------------------------------------

# Innovation throughput

# --------------------------------------------------------

innovation_scale = np.array([12.0, 8.0, 10.0, 6.0])

N = innovations_per_year(v, innovation_scale)

# --------------------------------------------------------

# Financials

# --------------------------------------------------------

rev_v = np.array([500_000, 150_000, 300_000, 200_000])

rev_n = np.array([80_000, 40_000, 100_000, 120_000])

base_cost = np.array([250_000, 120_000, 200_000, 180_000])

cost_v = np.array([180_000, 80_000, 140_000, 100_000])

pdca_p = np.array([20_000, 15_000, 30_000, 10_000])

pdca_d = np.array([30_000, 25_000, 35_000, 20_000])

pdca_i = np.array([40_000, 20_000, 45_000, 25_000])

R = revenue_function(v, N, rev_v, rev_n)

E_cost = expense_function(v, base_cost, pdca_p, pdca_d, pdca_i, cost_v)

ROI = roi(R, E_cost)

# --------------------------------------------------------

# Aggregate system metrics

# --------------------------------------------------------

v_sys_bottleneck = np.min(v, axis=1)

v_sys_mean = v @ alpha

N_total = np.sum(N, axis=1)

R_total = np.sum(R, axis=1)

E_total = np.sum(E_cost, axis=1)

ROI_total = R_total / np.maximum(E_total, 1e-12)

# --------------------------------------------------------

# Build dataframe

# --------------------------------------------------------

cols = {}

wheel_names = ["clinical", "education", "research", "governance"]

for j, name in enumerate(wheel_names):

cols[f"E_{name}"] = E[:, j]

cols[f"F_{name}"] = F[:, j]

cols[f"f_{name}"] = f[:, j]

cols[f"v_{name}"] = v[:, j]

cols[f"N_{name}"] = N[:, j]

cols[f"R_{name}"] = R[:, j]

cols[f"Ecost_{name}"] = E_cost[:, j]

cols[f"ROI_{name}"] = ROI[:, j]

df = pd.DataFrame({

"CTI_M": M,

"TEI_G": tei_g,

"TEI_B": tei_b,

"TEI": tei,

"v_sys_bottleneck": v_sys_bottleneck,

"v_sys_mean": v_sys_mean,

"N_total": N_total,

"R_total": R_total,

"E_total": E_total,

"ROI_total": ROI_total,

**cols,

})

# Simple interpretation band

df["LHS_state"] = pd.cut(

df["TEI"],

bins=[-np.inf, 0.6, 0.9, 1.2, np.inf],

labels=["Fragmented", "Emerging", "Functional", "Integrated"],

)

return df

# ============================================================

# 5) Example execution and summary

# ============================================================

if __name__ == "__main__":

df = simulate_lhs_drivetrain(n_projects=2000, seed=7)

print("\n--- Overall summary ---")

print(df[["CTI_M", "TEI_G", "TEI_B", "TEI", "v_sys_bottleneck", "ROI_total"]].describe().round(3))

print("\n--- LHS state counts ---")

print(df["LHS_state"].value_counts().sort_index())

print("\n--- Mean metrics by LHS state ---")

state_summary = df.groupby("LHS_state")[["CTI_M", "TEI_G", "TEI_B", "TEI", "v_sys_mean", "ROI_total", "N_total"]].mean()

print(state_summary.round(3))

# Example: CTI quartiles

df["CTI_quartile"] = pd.qcut(df["CTI_M"], 4, labels=["Q1_low", "Q2", "Q3", "Q4_high"])

quartile_summary = df.groupby("CTI_quartile")[["TEI_G", "TEI_B", "TEI", "ROI_total", "v_sys_bottleneck"]].mean()

print("\n--- Mean metrics by CTI quartile ---")

print(quartile_summary.round(3))
